# PDIA3P1 promotes Temozolomide resistance in glioblastoma by inhibiting C/EBPβ degradation to facilitate proneural-to-mesenchymal transition

**DOI:** 10.1186/s13046-022-02431-0

**Published:** 2022-07-15

**Authors:** Zijie Gao, Jianye Xu, Yang Fan, Yanhua Qi, Shaobo Wang, Shulin Zhao, Xing Guo, Hao Xue, Lin Deng, Rongrong Zhao, Chong Sun, Ping Zhang, Gang Li

**Affiliations:** 1 Department of Neurosurgery, Qilu Hospital of Shandong University, Cheeloo College of Medicine and Institute of Brain and Brain-Inspired Science, Shandong University, 107 Wenhua Western Road, Jinan, 250012 Shandong China; 2 Shandong Key Laboratory of Brain Function Remodeling, Jinan, 250012 Shandong China; 3 Tianjin Neurological Institute, Key Laboratory of Post-Neuroinjury Neuro-repair and Regeneration in Central Nervous System, Ministry of Education and Tianjin City, Tianjin Medical University General Hospital, Tianjin, China; 4 Immune Regulation in Cancer, Germany Cancer Research Center (DKFZ), 69120 Heidelberg, Germany

**Keywords:** PDIA3P1, Temozolomide, Proneural-to-mesenchymal transition, Glioma stem cells, C/EBPβ, MDM2, Neflamapimod

## Abstract

**Background:**

Resistance to temozolomide (TMZ) is a major obstacle to preventing glioblastoma (GBM) recurrence after surgery. Although long noncoding RNAs (lncRNAs) play a variety of roles in GBM, the lncRNAs that regulate TMZ resistance have not yet been clearly elucidated. This study aims to identify lncRNAs that may affect TMZ treatment sensitivity and to explore novel therapeutic strategies to overcome TMZ resistance in GBM.

**Methods:**

LncRNAs associated with TMZ resistance were identified using the Cancer Cell Line Encyclopedia (CCLE) and Genomics of Drug Sensitivity in Cancer (GDSC) datasets. Quantitative real-time PCR (qRT–PCR) was used to determine the expression of PDIA3P1 in TMZ-resistant and TMZ-sensitive GBM cell lines. Both gain-of-function and loss-of-function studies were used to assess the effects of PDIA3P1 on TMZ resistance using in vitro and in vivo assays. Glioma stem cells (GSCs) were used to determine the effect of PDIA3P1 on the GBM subtype. The hypothesis that PDIA3P1 promotes proneural-to-mesenchymal transition (PMT) was established using bioinformatics analysis and functional experiments. RNA pull-down and RNA immunoprecipitation (RIP) assays were performed to examine the interaction between PDIA3P1 and C/EBPβ. The posttranslational modification mechanism of C/EBPβ was verified using ubiquitination and coimmunoprecipitation (co-IP) experiments. CompuSyn was leveraged to calculate the combination index (CI), and the antitumor effect of TMZ combined with nefllamapimod (NEF) was validated both in vitro and in vivo.

**Results:**

We identified a lncRNA, PDIA3P1, which was upregulated in TMZ-resistant GBM cell lines. Overexpression of PDIA3P1 promoted the acquisition of TMZ resistance, whereas knockdown of PDIA3P1 restored TMZ sensitivity. PDIA3P1 was upregulated in MES-GBM, promoted PMT progression in GSCs, and caused GBMs to be more resistant to TMZ treatment. Mechanistically, PDIA3P1 disrupted the C/EBPβ-MDM2 complex and stabilized the C/EBPβ protein by preventing MDM2-mediated ubiquitination. Expression of PDIA3P1 was upregulated in a time- and concentration-dependent manner in response to TMZ treatment, and TMZ-induced upregulation of PDIA3P1 was mediated by the p38α-MAPK signaling pathway. NEF is a small molecule drug that specifically targets p38α with excellent blood–brain barrier (BBB) permeability. NEF blocked TMZ-responsive PDIA3P1 upregulation and produced synergistic effects when combined with TMZ at specific concentrations. The combination of TMZ and NEF exhibited excellent synergistic antitumor effects both in vitro and in vivo.

**Conclusion:**

PDIA3P1 promotes PMT by stabilizing C/EBPβ, reducing the sensitivity of GBM cells to TMZ treatment. NEF inhibits TMZ-responsive PDIA3P1 upregulation, and NEF combined with TMZ provides better antitumor effects.

**Supplementary Information:**

The online version contains supplementary material available at 10.1186/s13046-022-02431-0.

## Introduction

Glioblastoma multiforme (GBM) is the most common malignant and aggressive tumor of the central nervous system (CNS) [1, 2]. Almost all GBM patients experience relapse despite the usual combination of surgery, chemotherapy and radiation therapy, and the median survival time has been approximately 12 to 15 months for decades [3, 4]. Obstacles to glioma treatment are due not only to the limited extent of the tumor that can be safely removed but also to resistance to adjuvant therapy after surgical resection [5]. Temozolomide (TMZ), a second-generation oral alkylating agent, is the first-line chemotherapeutic agent for patients with GBM [6, 7]. However, nearly all patients develop resistance to TMZ and relapse after a progression-free survival period of 7 to 10 months [8]. Therefore, it is urgent to elucidate the underlying mechanisms of TMZ resistance to treat and prevent GBM recurrence.

Long non-coding RNAs (LncRNAs) is a class of heterogeneous RNA that are more than 200 nucleotides in length and limit protein coding potential [9]. LncRNAs have been proven to perform diverse cellular functions, including transcriptional regulation in cis or trans, organization of nuclear domains, and posttranscriptional regulation by interacting with miRNAs, mRNAs, or proteins [10–12]. Emerging evidence has shown that lncRNAs are associated with multiple features of cancer, such as proliferation, apoptosis, metastasis, metabolism, and therapy resistance [13, 14]. Recent studies have demonstrated that lncRNAs regulate numerous signaling pathways through interactions with proteins [15–17]. However, the regulation of posttranslational modifications by lncRNAs and the subsequent impact on TMZ treatment resistance in GBM remain largely uncharacterized.

Various mechanisms contribute to TMZ resistance in GBM, of which GBM cell heterogeneity and plasticity are thought to be key factors driving treatment resistance and tumor recurrence [18]. Based on bulk RNA sequencing findings, intertumor heterogeneity is manifested by at least three GBM subtypes, including proneural (PN), classical (CL) and mesenchymal (MES) [19]. Heterogeneity is also manifested by differences in the developmental status of GBM cells in tumors. Glioma stem cells (GSCs) are a group of cells with the capacity for self-renewal and asymmetric differentiation [20]. The presence of GSCs is thought to be a driving force in tumorigenesis, tumor propagation and preferential resistance to radiotherapy and chemotherapy; thus, GSCs are considered a valuable model for studying GBM [21]. GBMs of PN and MES subtypes correspond to PN and MES GSCs, respectively, but no GSCs corresponding to the CL subtype of GBM have been identified [22]. Recent studies have shown that GBM undergoes proneural-to-mesenchymal transition (PMT) as the disease progresses and the tumor recurs [23–25]. PMT is therefore considered a marker of tumor tolerance in response to multiple treatments and tumor recurrence. A variety of possible mechanisms drive the occurrence of PMT, including intracellular signaling pathways and the extracellular tumor microenvironment (TME). For instance, Carro et al. identified STAT3 and C/EBPβ as two master regulators (MRs) of PMT [26]. In addition, the impact of treatment and the subsequent selective pressure within the tumor may contribute PMT [27]. However, the mechanisms of treatment-induced PMT and modulation of MRs by lncRNAs remain unclear.

In this study, we identified a key lncRNA, PDIA3P1, which is closely associated with GBM TMZ therapy resistance and recurrence. In vitro and in vivo assays revealed that knockdown of PDIA3P1 resulted in decreased resistance to TMZ in glioma cells; in contrast, overexpression of PDIA3P1 resulted in increased resistance of glioma cells to TMZ. Mechanistically, PDIA3P1 promoted PMT by stabilizing CEBPβ, enabling GSCs to acquire preferential resistance to TMZ treatment. Even more valuable, we identified a drug called neflamapimod (NEF) that specifically targets p38α and has the ability to easily cross the blood–brain barrier (BBB). We demonstrated that NEF inhibits TMZ-induced upregulation of PDIA3P1 and enhances the sensitivity of glioma cells to TMZ treatment.

## Materials and methods

### Public data collection

TMZ sensitivity data from GBM cell lines were obtained from the GDSC (www.cancerRxgene.org) database which is the largest public database on molecular markers of cancer drug sensitivity and drug response [28]. Corresponding cell lines expression data were available from the CCLE (https://portals.broadinstitute.org/ccle/) [29]. Transcript level data, somatic mutation and associated clinical information of TCGA GBM were extracted from GDC Data Portal (https://portal.gdc.cancer.gov/). The RNA-seq transcriptome data and clinical traits of the CGGA GBM were downloaded from CGGA database (http://www.cgga.org.cn/). The microarray information of GSC expression was available in the Gene Expression Omnibus (GEO) database (GSE68029 at www.ncbi.nlm.nih.gov/geo).

### Differential expression analysis

The limma R package was leveraged to identify differentially expressed genes (DEGs) between TMZ resistance and sensitive cell lines. The top 30 upregulated genes sorted according to *p*-value in TMZ resistant group were visualized using the pheatmap R package.

### Single sample gene set enrichment analysis (ssGSEA)

To determine the abundance of GBM immune infiltration levels, immune gene signatures were obtained from data of Bindea et al. [30] to perform ssGSEA. The immune cell infiltration levels were estimated using “GSVA” R package based on deconvolution algorithm.

### Gene set enrichment analysis (GSEA)

The gene sets of “c2.cp.kegg.v7.4”, “c5.go.bp.v7.4”, “verhaak glioblastoma mesenchymal” and “verhaak glioblastoma proneural” were obtained from The Molecular Signatures Database (MSigDB; http://www.gsea-msigdb.org/gsea/login.jsp) database for running GSVA. *P*-value < 0.05 indicates statistical significance.

### Cell lines and cell culture

Human glioma cell lines U118MG, U87MG, LN229 and U251MG were purchased from the Chinese Academy of Sciences Cell Bank and cultured in DMEM medium (Gibco, USA) with 10% fetal bovine serum (FBS). The neural progenitor cell (NPC) and GBM patient-derived GSC cell lines and were kindly donated by Dr. Krishna P.L. Bhat (The University of Texas, M.D. Anderson Cancer Center, Houston, TX). GSC lines (GSC20, GSC267, GSC8–11, GSC11) have been used extensively in previous studies and the subtypes of GSCs have been clarified according to the Verhaak or Philips gene signatures, respectively. GSCs and NPC were cultured in DMEM/F12 (Gibco, USA) with 2% B-27 no serum supplement (Gibco, USA), 20 ng/mL human recombinant bFGF (R&D Systems) as well as 20 ng/mL human recombinant EGF (R&D Systems, USA). The GSC or NPC spheres were digested using accutase solution (Sigma-Aldrich, USA). All cell lines were cultured in a humid chamber at 37 °C and containing 5% carbon dioxide and 5% oxygen.

### RNA extraction and quantitative real-time PCR (RT-qPCR)

TRIzol (Invitrogen, USA) was used to extract total RNA according to manufacturer’s instruction. The high capacity cDNA Reverse Transcription Kit (Toyobo, China) was leveraged for reverse transcription in accordance with the manufacturer’s protocol. An Mx-3000P Quantitative PCR System (Applied Biosystems, USA) was used for qRT-PCR. The primers used for RT-qPCR were: 5′-GGAAAACCACTGGGGAGGAC-3′ (forward) and 5′-CAGTGCAGCTAAGAAATGGCT-3′ (reverse) for PDIA3P1; 5′-GCACCGTCAAGGCTGAGAAC-3′ (forward) and 5′-TGGTGAAGACGCCAGTGGA-3′ (reverse) for GAPDH; 5′-TTTGTCCAAACCAACCGCAC-3′ (forward) and 5′-GCATCAACTTCGAAACCGGC-3′ (reverse) for CEBPB.

### Plasmids, viral transfections and cloning

Human full-length PDIA3P1 as well as sh-PDIA3P1 plasmids were used in the current study for stable overexpression and knockdown, respectively, whereas empty plasmid was used as a control. Lentiviral particles were constructed by transfecting 293 T cells with the packaging vectors psPAX2 and pMD2G. Lentiviral particles were collected 24 and 48 hours after transfection of 293 T cells, filtered through a 0.45 μm filter (Corning), and then used to treat cells in culture. After 48 hours, cells were selected by Puromycin (2 μg/mL). All small interfering RNAs (siRNA) and overexpression plasmids were purchased from Genepharma (shanghai, China). For short-term knockdown and overexpression of GBM cells, cells were transfected of siRNAs and plasmids using the Lipofectamine 3000 kit (Invitrogen, USA) according to the manufacturer’s instruction.

### Reagents and antibodies

TMZ and NEF (Synonyms: VX-745) were purchased from MedChemExpress (MCE, https://www.medchemexpress.cn/). TMZ and NEF were dissolved in dimethyl sulfoxide (DMSO) at concentrations of 100 mM and 10 mM, respectively. TMZ and NEF in solvent are stored at − 20 °C and used up within 1 month. The primary antibodies used in this study are listed as follows: β-actin (Cell Signaling Technology, 8480), CD44 (Cell Signaling Technology, 3570), C/EBPβ (Abcam, ab32358), YKL-40 (Cell Signaling Technology, 47,066), SOX2 (Cell Signaling Technology, 3579), γ-H2AX (Cell Signaling Technology, 7631), MDM2 (Abcam, ab259265), JUN (Cell Signaling Technology, 9165), p-JUN (Cell Signaling Technology, 3270), ubiquitin (Cell Signaling Technology, 3933), P38 (Cell Signaling Technology, 8690), p-P38 (Cell Signaling Technology, 4511).

### CCK-8 assay and drug treatment

CCK-8 reagent (RiboBio, China) was used to assess GBM cells viability. We seeded GBM cells in 96-well plates at a density of 2 × 10^3^ cells per well in 100 μl of Gibco DMEM containing 10% FBS. The cells were incubated at 37 °C 12 h for cells adhesion and then treated with different concentrations of TMZ or NEF. After incubation for 48 h, 10 μl of CCK-8 solution was added to each well for 1 h before measurement. Absorbance (OD value) at 450 nm was measured using a microplate.

### Alkaline comet assay

The alkaline comet assay was used to detect the damaged DNA with high sensitivity [31]. GBM cells in different groups were harvested in PBS at a 1–3*10^5^ cells/ml density. Cells were mixed with molten LM agarose at a ratio of 1:10 (V/V) and 50 μl of the mixture was immediately pipetted onto a CometSlide. Then cells were lysed in alkaline lysis solution at 4 °C for 12 h for lysis. After that, the slides were soaked with alkaline electrophoresis buffer for 20 minutes away from light and electrophoresis for 30 min at 25 V. After precipitation and washing, the slides were stained with Green-DNA Dye and images were captured by fluorescence microscopy.

### Immunofluorescence (IF) assay

GBM cells were fixed in 4% paraformaldehyde for 15 min and washed three times in PBS. Then cells were permeabilized in 0.3% Triton X-100 for 10 min and blocked with 5% Goat serum for 1 h. Then the cells were incubated with indicated primary antibodies overnight at 4 °C. Cells were then incubated with fuorescent second antibody at room temperature for 1 h. DAPI was used to counterstain nuclei for 15 min. Images were captured using a LeicaSP8 confocal microscope.

### EdU assay

EdU cell proliferation assay kit (RiboBio, China) was used to determine cell proliferation. Cells were incubated with 200 μl of 5-ethynyl-20-deoxyuridine at 37 °C for 2 h. After fixed and permeabilized, the cells were incubated with Apollo reagent for 30 min and the Hoechst were used to stain nuclei. The images were viewed and obtained using fluorescence microscope.

### Flow cytometry

Both suspended and adherent GBM cells were obtained for apoptosis analysis after treating with TMZ or DMSO (solvent control of TMZ) for 48 h. Annexin VFITC and PI staining (BD Biosciences, USA) was leveraged for apoptosis analysis according to the instruction. The number of cells were counted by BD Accuri C6 flow cytometer.

### Colony formation assay

We seeded about 2000 GBM cells in 6-well plates per well in 1.5 ml of Gibco DMEM containing 10% FBS. The cells were incubated in a humidified chamber containing 5% CO2 and 5% O2 at 37 °C for 2 weeks. After that, colonies were fixed and stained with crystal violet (Solarbio, China) for 20 min. The colonies were washed with PBS for at least three times and the number of colonies were counted.

### Neurosphere formation assay

We seeded about 1000 GSCs per well in 6-well plates with 1.5 ml DMEM/F12 containing 2% B-27. After 7 days incubation at 37 °C, the images were acquired and the relative diameters of neurospheres were calculated.

### Extreme limiting dilution assay (ELDA)

We implanted GSCs into ultralow-attachment 96-well plates at densities of 0, 2, 4, 8, 16, 32, 64 and 128 cells per well in 10 replicates. The number of wells with neurospheres formation was counted after 7 days incubation. Collected data was analyzed using (http://bioinf.wehi.edu.au/software/elda/).

### Protein half-life assay

CHX was used to inhibit new proteins synthesis. Cells were treated with 100 μg/ml CHX for 0 h, 2 h, 4 h, 6 h and 8 h prior to protein collection. The proteins levels were detected by western blot assay.

### RNA pull-down assay and RNA immunoprecipitation (RIP) assay

Biotinylated PDIA3P1 and its anti-sense sequence were synthesized by RiboBio (GenePharma, China). Pierce™ Magnetic RNA-protein pull-down kit (Thermo Fisher Scientific, SA) was used for RNA pull-down assay. Cell lysates of GSC267 were firstly incubated with a biotin-labelled PDIA3P1 probe. Then the conjugated magnetic beads were added to cell lysates and the interacting proteins were separated by western blot and then the silver staining was used for visualization.

Magna RIP kit (Millipore, USA) was leveraged for RIP assay according to manufacturer’s instruction. RT-qPCR was used for detecting the relative expression of immunoprecipitated RNA. The IgG antibody (from Magna RIP kit) was used for negative control.

### Immunoprecipitation (IP) assay

The IP assay was performed using Pierce Classic Magnetic immunoprecipitation (IP)/Co-IP Kit (Thermo Fisher, USA) according to the manufacturer’s instruction. Firstly, the different antibodies were incubated with protein A/G magnetic beads. Then the cell lysates from GSCs were collected and incubated with antibody coupled beads. The beads interacting proteins were washed and denatured and the proteins were examined using western blotting.

### Drug combination analysis

To assess the combination of effect of TMZ and NEF, GBM cells were treated with different concentrations of TMZ and NEF for 48 h in 3 replicates. CompuSyn software (Biosoft, Ferguson, MO, USA) was leveraged to evaluate drug synergism. The combination index (CI) values were calculated using non-constant ratios drug combination analysis according to instruction of the software. CI < 0.75, CI = 0.75–1.25, and CI > 1.25 were defined as synergistic, additive, and antagonistic effects, respectively.

### Animal studies

Luciferase labeled and stably transfected sh-PDIA3P1-U118MG cells or sh-Control-U118MG, or ov-PDIA3P1-U251 or ov-vector-U251 were injected into the brains of randomly grouped 4-week male BALB/c nude mice (5 × 10^5^ cells/mouse). On the fifth postoperative day, the mice were randomly divided into TMZ treatment or control groups. Mice were treated with or without TMZ by oral gavage per week (5 mg/kg, p.o., 5 times per week). For evaluating the anti-tumor effect of TMZ in combination with NEF in vivo, luciferase-labeled GSC267 cells (1 × 10^6^ cells/mouse) were implanted into the brains of 4-week male BALB/c nude mice. After 7 days post-operative, the mice were randomly divided into four groups, control, TMZ only (5 mg/kg, p.o.,5 times per week), NEF only (5 mg/kg, p.o.,5 times per week) and combination group. To evaluate the intracranial tumor, bioluminescence imaging was used to quantify tumor burden using an IVIS Lumina Series III (PerkinElmer). All procedures used for animal treatments and experiments were approved by and under the requirements of the Animal Care and Use Committee of the Qilu Hospital of Shandong University.

### Statistical analysis

All statistical analysis was conducted by R 4.1.1 and GraphPad Prism 8.0 software. Acquired data were certified as normal distribution through Shapiro-Wilk Normality test and homogeneity of variances through Bartlett test. Then t-tests and one-way ANOVA were used for comparisons between two independent samples and comparisons among multiple samples, respectively. The Wilcoxon test was used for non-parametric data. *P*-value < 0.05 was considered statistically significant (*p-value < 0.05; **p-value < 0.01; ***p-value < 0.001). The receiver operating characteristic (ROC) curve was used to evaluate the diagnostic value of PDIA3P1, and the area under the curve (AUC) was quantified using the pROC R package. Pearson correlation was used to calculate the correlation between two or more groups. Kaplan-Meier curve and log-rank test were used to evaluate survival between different groups.

## Results

### PDIA3P1 is upregulated in TMZ-resistant cell lines and promotes TMZ resistance

**Fig. 1:**
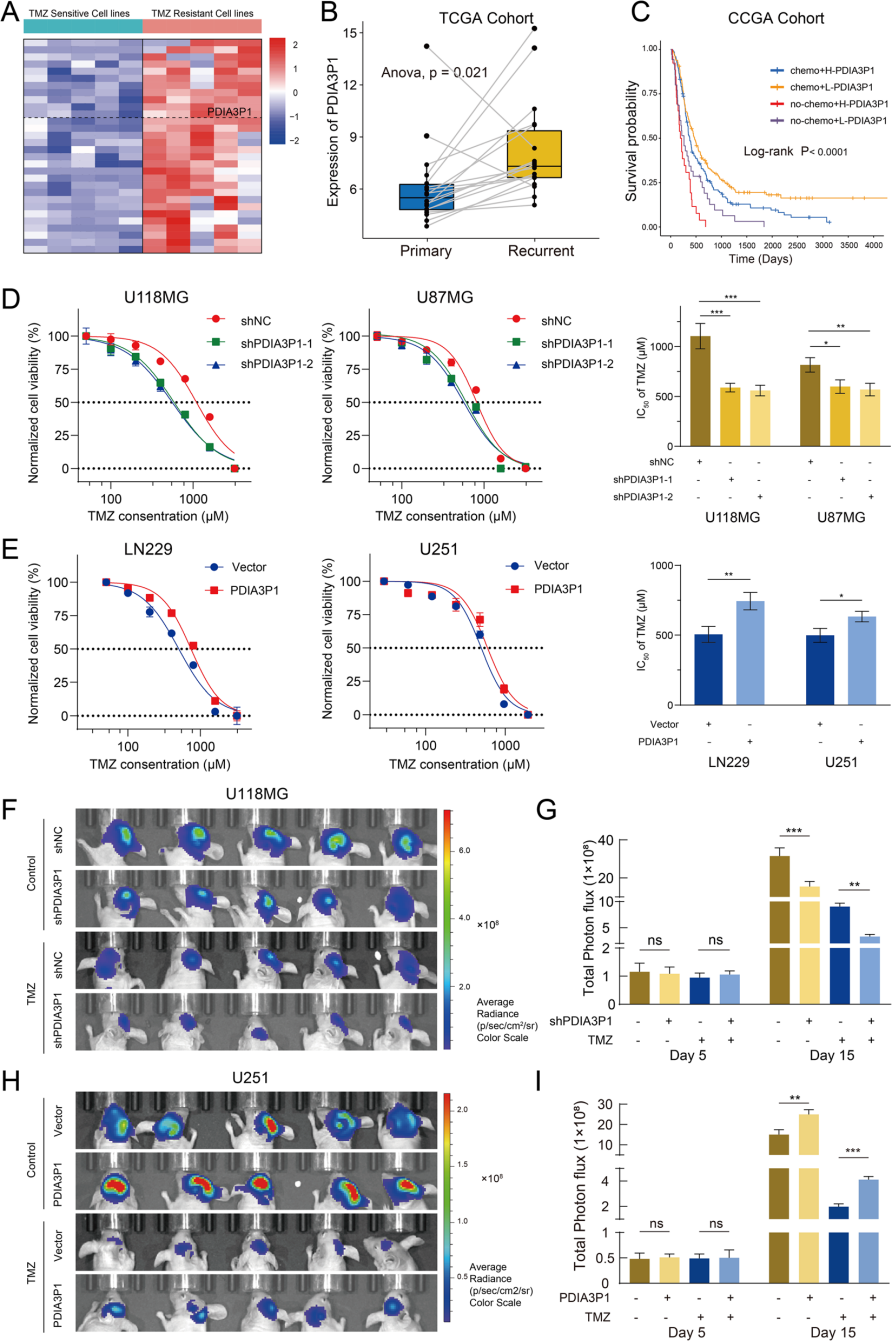
PDIA3P1 is upregulated in TMZ-resistant cell lines and promotes TMZ resistance. **A** Heatmap showed that PDIA3P1 was upregulated in TMZ resistance cell lines. **B** The expression of PDIA3P1 is higher in primary gliomas than in recurrent gliomas. **C** Survival analysis of GBM patients stratified by whether they received chemotherapy and expression level of PDIA3P1. **D** Cell viability assay of PDIA3P1-knockdown and control U118MG and U87MG cells treated with various concentrations of TMZ for 48 h. The detailed IC_50_ were listed in the right panel. **E** Cell viability assay of PDIA3P1-overexpression and control LN229 and U251 cells treated with various concentrations of TMZ for 48 h. The detailed IC_50_ were listed in the right panel. **F H** Bioluminescence imaging of tumor growth in xenograft nude mice with PDIA3P1 knockdown (**F**) or overexpression (**H**) and receiving or exempt from TMZ treatment in U118MG and U251 xenografts, respectively. **G I** The quantification of the photon counts of U118MG and U251 xenografts, respectively. The tumor sizes were monitored on day 5 and day 15

To identify potential lncRNAs involved in GBM resistance to TMZ chemotherapy, information for 10 glioma cell lines paired with specific half-maximal inhibitory concentration (IC_50_) values of TMZ were obtained from GDSC, and RNA-seq data from corresponding cell lines were downloaded from CCLE. We divided the glioma cell lines into TMZ-resistant and TMZ-sensitive groups based on IC_50_ values. The limma package was utilized to analyze DEGs between the two groups. The clustered heatmap (Fig. 1A) shows the top 30 upregulated genes in the TMZ-resistant group sorted according to *p* value. Among the DEGs, PDIA3P1 was markedly upregulated in TMZ-resistant cell lines (log_2_Fold change = 1.6, *P* < 0.001) (Fig. S1A and Table. S1). Based on bioinformatics analysis, we found that expression of PDIA3P1 was higher in recurrent gliomas than in primary gliomas (Fig. 1B). Survival analysis revealed that higher PDIA3P1 levels were related to poorer progression-free survival (PFS) in GBM patients (Fig. S1B). In addition, the low-PDIA3P1 group exhibited a significant survival advantage in GBM patients either receiving or not receiving chemotherapy (Fig. 1C). Next, qRT–PCR on two TMZ-sensitive (U251: IC_50_ = 497.9 μM; LN229: IC_50_ = 503.0 μM) and TMZ-resistant (U118MG: IC_50_ = 1100.0 μM; U87MG: IC_50_ = 814.7 μM) GBM cell lines verified that PDIA3P1 was upregulated in TMZ-resistant cell lines (Fig. S1C).

To investigate the functional role of PDIA3P1 in promoting TMZ resistance, PDIA3P1 was knocked down using two independent shRNAs in U118MG and U87MG cells and overexpressed in U251 and LN229 cells. The expression of PDIA3P1 was detected using qRT–PCR (Fig. S1D). Knockdown of PDIA3P1 in resistant cell lines (U118MG and U87MG) resulted in a notable reduction in IC_50_ and further inhibition of the tumor cell growth rate upon TMZ treatment (Fig. 1D and Fig. S1E). In contrast, overexpression of PDIA3P1 in sensitive cell lines (U251 and LN229) resulted in a significant increase in IC_50_ values and counteracted the inhibitory effect of TMZ on tumor cell growth (Fig. 1E and Fig. S1E).

To evaluate the effect of PDIA3P1 on the TMZ-resistant phenotype in vivo, 5 × 10^5^ luciferase-labeled U118MG-shPDIA3P1 or U118-shNC and U251-PDIA3P1 or U251-Vector cells were injected into nude mice. We tracked tumor proliferation using in vivo bioluminescence imaging. Despite the initial tumor size being similar (Fig. S2A, B), xenografts bearing U118MG-shPDIA3P1 cells displayed significant tumor growth inhibition, whereas xenografts bearing U251-PDIA3P1 cells exhibited tumor growth promotion. As expected, TMZ treatment (5 mg/kg, p.o., 5 times per week) reduced tumor burden. Tumor size in the U118MG-shPDIA3P1 group was reduced compared to that in the control group (Fig. 1F, G), while tumor size in the U251-PDIA3P1 group was relatively increased compared to that in the control group (Fig. 1H, I). Consistently, Kaplan–Meier curves demonstrated that the overall survival time of mice was prolonged in the PDIA3P1 knockdown group with and without TMZ treatment (Fig. S2C). Although TMZ treatment significantly prolonged the survival time of mice in the U251-Vector group, PDIA3P1 overexpression decreased the survival time of mice in both the treatment and control groups (Fig. S2D). H&E-stained mouse brain sections showed that knockdown of PDIA3P1 greatly reduced tumor invasiveness, with or without TMZ treatment, whereas overexpression of PDIA3P1 promoted tumor invasiveness (Fig. S2E, F). Taken together, these findings indicate that PDIA3P1 promotes glioma cell resistance to TMZ both in vitro and in vivo.

### The effect of PDIA3P1 on TMZ treatment-induced DNA damage and inhibition of proliferation

**Fig. 2:**
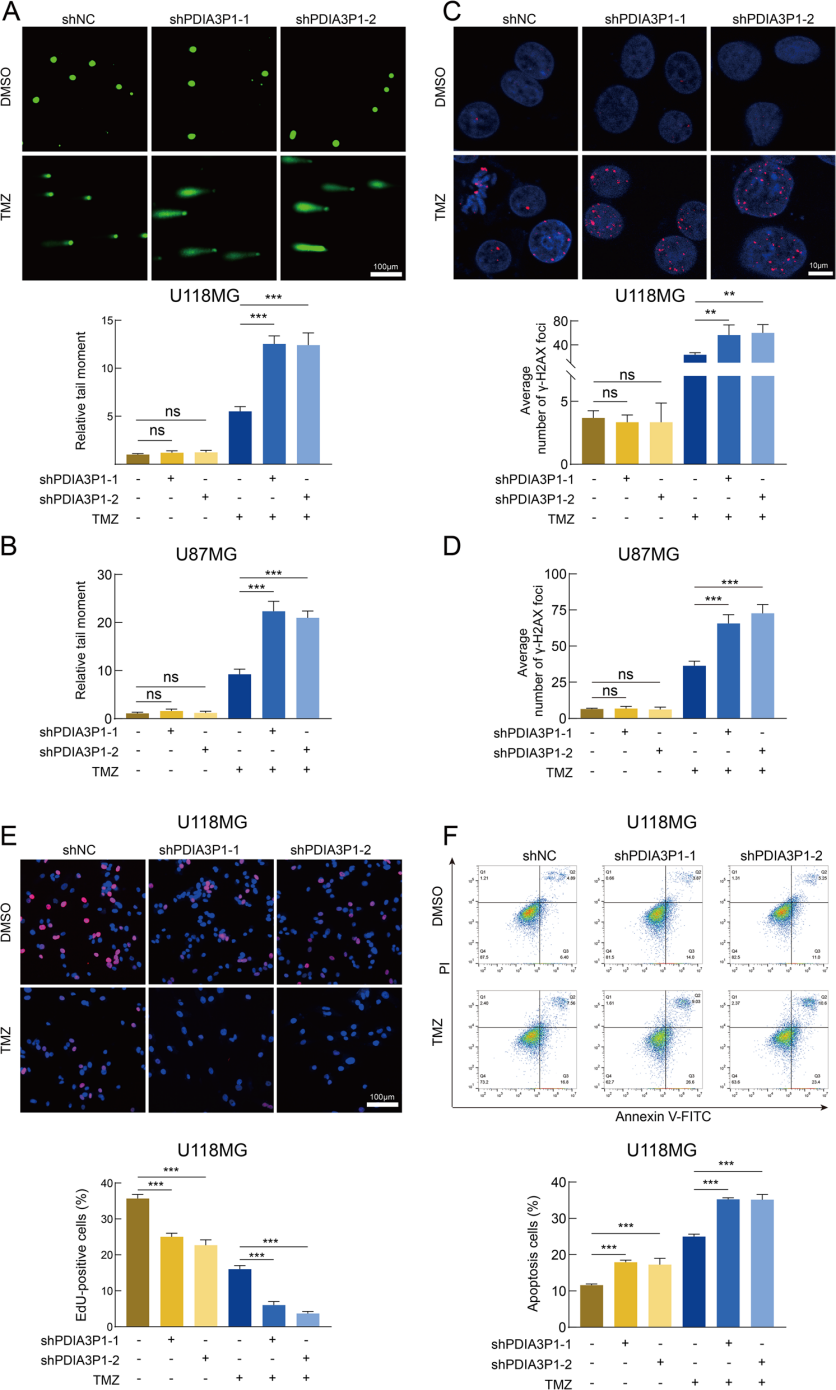
Knockdown of PDIA3P1 exacerbates DNA damage and proliferation inhibition induced by TMZ intervention. **A B** Representative images and quantification of comet assay showing the DNA damage caused by PDIA3P1 knockdown or vehicle control with or without TMZ treatment (400 μM, 48 h) on U118MG (**A**) and U87MG (**B**) cells. Scale bars, 100 μm. **C D** Representative images and quantification of γ-H2AX staining on U118MG (**C**) and U87MG (**D**) cells with or without TMZ treatment (400 μM, 48 h). Scale bars, 10 μm. **E** Representative images of U118MG cells subjected to the EdU cell proliferation assay (upper panel; scale bar, 100 μm) and quantification of EdU-positive cells (lower panel) with or without TMZ treatment (400 μM, 48 h). **F** Apoptosis assay showing the effect of PDIA3P1 knockdown on U118MG with or without TMZ treatment (400 μM, 48 h). The lower panel was the quantification of apoptosis cells

To explore the biological behaviors of PDIA3P1, we performed GSVA enrichment. The high PDIA3P1 expression group was significantly enriched in damage repair and stress response pathways, such as the regulation of DNA repair and cellular response to chemical stress, suggesting that PDIA3P1 may play a role in damage repair and the stress response (Fig. S3A and Table. S2). In addition, the PDIA3P1 high expression group exhibited a lower frequency of IDH1 mutation (Fig. S3C). Because TMZ exerts its antitumor effects primarily by damaging DNA and inducing programmed cell death (PCD), we performed comet and ɣH2AX IF assays to assess DNA damage. The alkaline comet assay is a sensitive method to detect DNA double-strand breaks (DSBs) and single-strand breaks. We observed increased DNA damage in shPDIA3P1 cells after TMZ treatment, whereas knockdown of PDIA3P1 had very little effect on DNA damage in the absence of TMZ in U118MG and U87MG cells (Fig. 2A, B and Fig. S4A). Phosphorylated histone H2AX (ɣH2AX) is an indicator of the DNA damage response (DDR). When DNA damage occurs, ɣH2AX can be recruited to lesions [32]. Using an IF staining assay, we confirmed that knockdown of PDIA3P1 increased nuclear ɣH2AX levels in response to TMZ treatment, while nuclear ɣH2AX levels remained virtually unchanged in the absence of TMZ intervention (Fig. 2C, D and Fig. S4B). We further performed EdU, colony formation and apoptosis assays to explore the function of PDIA3P1. EdU and colony formation assays revealed that knockdown of PDIA3P1 inhibited cell proliferation, whereas with respect to TMZ treatment, knockdown of PDIA3P1 inhibited cell growth even more (Fig. 2E and Fig. S4C, D). Next, we assessed the apoptosis rate using flow cytometry, and the proportion of apoptotic cells significantly increased in the PDIA3P1 knockdown group after TMZ treatment (Fig. 2F).

**Fig. 3:**
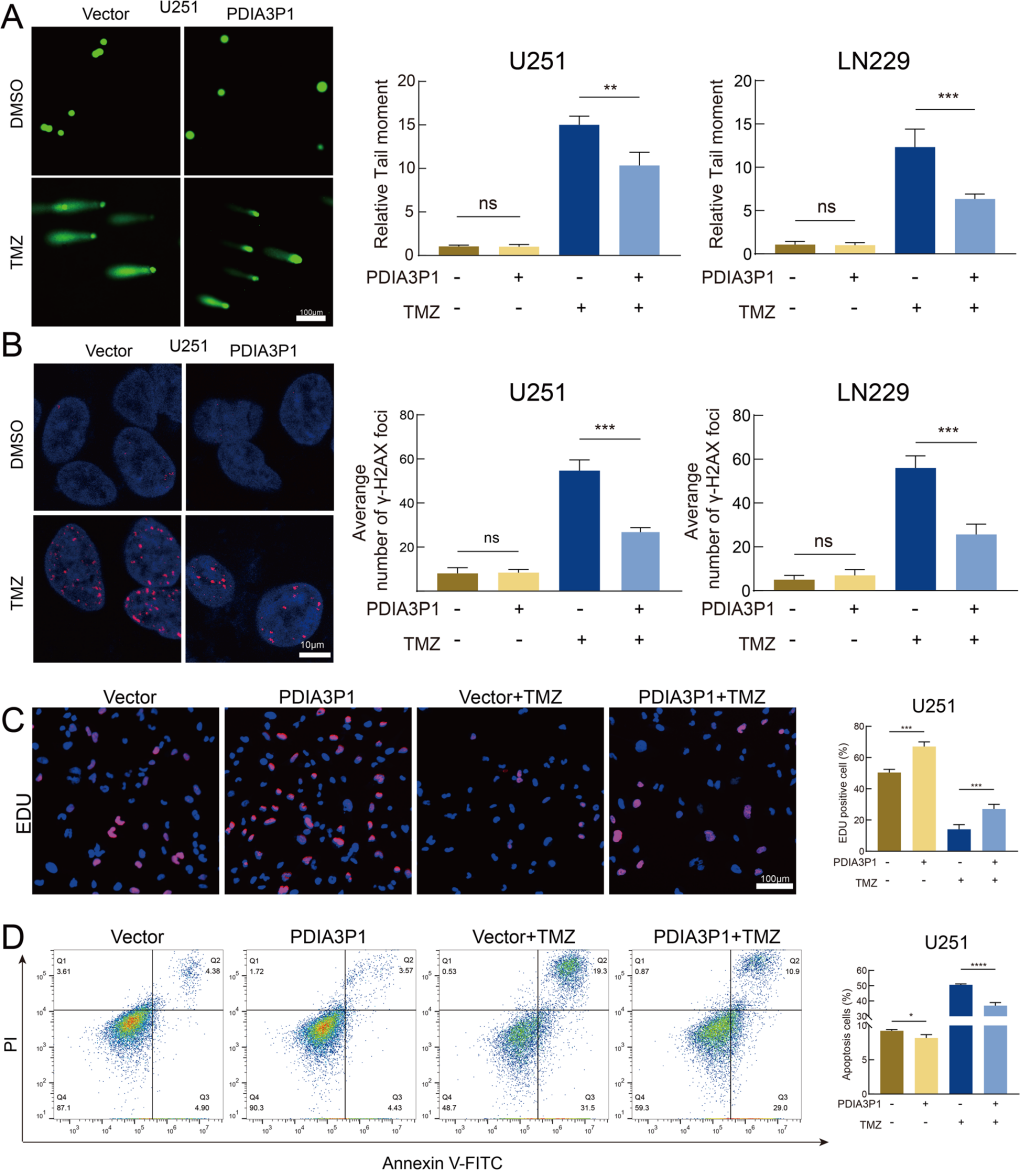
Overexpression of PDIA3P1 counteracted TMZ treatment-induced DNA damage and growth inhibition. **A** Representative images and quantification of comet assay on ov-PDIA3P1 or Vector U251 and LN229 cells with or without TMZ treatment (400 μM, 48 h). Scale bar, 100 μm. **B** Representative images and quantification of γ-H2AX staining on ov-PDIA3P1 or Vector U251 and LN229 cells with or without TMZ treatment (400 μM, 48 h). Scale bar, 10 μm. **C** Representative images of U251 cells subjected to the EdU cell proliferation assay (left panel; scale bar, 100 μm) and quantification of EdU-positive cells (right panel) with or without TMZ treatment (400 μM, 48 h). **D** Apoptosis assay showing the effect of PDIA3P1 overexpression on U251 with or without TMZ treatment (400 μM, 48 h). The right panel was the quantification of apoptosis cells

Since knockdown of PDIA3P1 was able to restore the sensitivity of GBM cells to TMZ, we investigated whether overexpression of PDIA3P1 could promote TMZ resistance. The comet assay showed that PDIA3P1 overexpression without TMZ intervention had little effect on DNA damage, while overexpression of PDIA3P1 salvaged TMZ-induced DNA damage (Fig. 3A and Fig. S4E). Similarly, IF staining assays demonstrated that PDIA3P1 overexpression decreased nuclear ɣH2AX levels after TMZ treatment, whereas nuclear ɣH2AX levels remained unchanged and at relatively low levels in the absence of TMZ (Fig. 3B and Fig. S4F). The EdU assay showed that overexpression of PDIA3P1 had a positive effect on cell proliferation. Furthermore, PDIA3P1 overexpression partially counteracted TMZ-mediated cell growth inhibition (Fig. 3C). We then evaluated the effect of PDIA3P1 overexpression on the apoptosis rate of GBM cells. As shown in Fig. 3D, a slight decrease in the apoptosis rate was observed in cells overexpressing PDIA3P1 compared to control cells without TMZ treatment, whereas PDIA3P1 overexpression greatly counteracted the apoptosis induced by TMZ treatment (Fig. 3D). To exclude the possibility that the observed function of PDIA3P1 is limited to cell lines, we performed further validation using GSC20, which is isolated from a GBM patient derived tumor. The comet assay and γ-H2AX IF staining assay demonstrated that knockdown of PDIA3P1 in GSC20 promoted the DNA damage induced by TMZ treatment (Fig. S5A, C), whereas overexpression of PDIA3P1 in GSC20 counteracted the DNA damage induced by TMZ treatment (Fig. S5B, D). The EdU assay showed that knockdown of PDIA3P1 inhibited GSC20 proliferation, whereas in the case of TMZ treatment, knockdown of PDIA3P1 in GSC20 inhibited cell growth even more (Fig. S5E). On the contrary, overexpression of PDIA3P1 in GSC20 promoted cell proliferation, while overexpression of PDIA3P1 in GSC20 partially counteracted TMZ-mediated cell growth inhibition (Fig. S5F). Hence, through this series of experiments, we demonstrated that knockdown of PDIA3P1 exacerbated TMZ intervention-induced DNA damage and growth inhibition, whereas overexpression of PDIA3P1 reduced DNA damage and proliferation inhibition caused by TMZ intervention.

### Elevated expression of PDIA3P1 is associated with the mesenchymal subtype of GBM

**Fig. 4:**
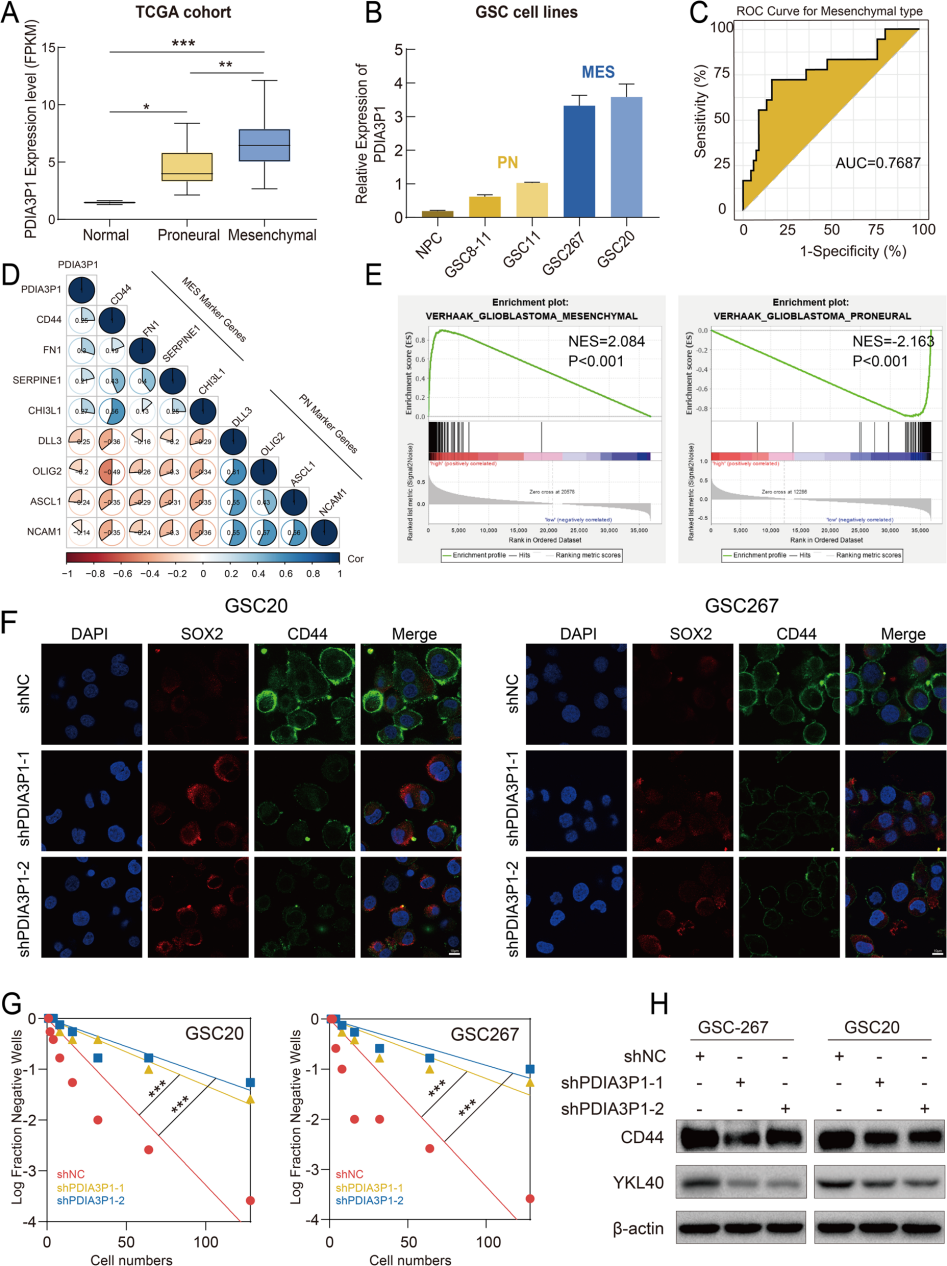
Elevated expression of PDIA3P1 is associated with Mesenchymal subtype. **A** Statistical analysis of PDIA3P1 in normal, proneural (PN) and mesenchymal (MES) tissues in the TCGA GBM dataset. **B** The relative expression of PDIA3P1 in NPC, PN GSCs and MES GSCs. **C** ROC curves of PDIA3P1 for MES-GBM subtype prediction in TCGA. **D** The PDIA3P1 expression is negatively correlated with PN related genes and positively correlated with MES related genes. **E** GSEA showed a significant positive correlation between the expression of PDIA3P1 and MES subtypes, and a negative correlation with PN subtypes. **F** Representative images of IF staining revealing the effect of PDIA3P1 knockdown on the expression of CD44 and SOX2 in GSC20 and GSC267, respectively. Scale bar, 10 μm. **G** Extreme limit dilution assays showing a decreased self-renewal ability after knockdown of PDIA3P1 in GSC20 and GSC267, respectively. **H** The protein expression of MES markers after PDIA3P1 knockdown in GSC20 and GSC267

We further investigated the mechanism of PDIA3P1-mediated TMZ resistance. Phenotypic heterogeneity and plasticity in GBM drive therapeutic resistance and recurrence. Compared to the PN subtype, which has a better survival prognosis and is sensitive to TMZ treatment, the MES subtype exhibits increased resistance to radiotherapy and chemotherapy and a higher risk of recurrence [25, 33]. We hypothesized that the function of PDIA3P1 in promoting TMZ resistance is mediated by affecting the GBM subtype. We first examined expression of PDIA3P1 in TCGA and CGGA datasets and found that PDIA3P1 expression was significantly higher in the MES subtype than in the PN subtype (Fig. 4A and Fig. S6A). The existence of GSCs is an important factor contributing to GBM heterogeneity and TMZ resistance, and GSCs are a valuable experimental model for GBM analysis. Detecting PDIA3P1 expression by qRT–PCR, we observed that PDIA3P1 expression was markedly upregulated in MES GSCs (GSC20, GSC267) compared to PN GSCs (GSC8–11, GSC11), and PDIA3P1 was least expressed in neuronal precursor cell lines (NPCs) (Fig. 4B). To explore the predictive efficiency of PDIA3P1 for GBM subtype, the area under the receiver operating characteristic (ROC) curve (AUC) was calculated, and PDIA3P1 expression was appropriate for assessing GBM subtypes (Fig. 4C and Fig. S6B). Meanwhile, we performed Pearson correlation analysis of gene expression and identified a significant positive correlation between PDIA3P1 and MES subtype-related genes (CD44, FN1, CHI3L1, SERPINE1), while PDIA3P1 was negatively correlated with PN subtype-related genes (DLL3, NCAM1, ASCL1, OLIG2) (Fig. 4D). We further performed GSEA of the relationship between PDIA3P1 and GBM subtypes based on the TCGA dataset. The results showed that the MES GBM subtype was enriched in the high PDIA3P1 expression group, whereas the PN GBM subtype was enriched in the low PDIA3P1 expression group (Fig. 4E). Two independent shRNAs were next transfected into GSC20 and GSC267 cells to investigate the causal relationship between PDIA3P1 and the GBM subtype (Fig. S6C). Stable knockdown of PDIA3P1 in GSC20 and GSC267 cells resulted in an obvious inhibition of tumorsphere expansion (Fig. S6D) and reduced sphere formation ability (Fig. 4G). These results demonstrated that PDIA3P1 is associated with the tumorigenesis and stemness of GSCs. CD44 and SOX2 are protein markers of the MES and PN subtypes, respectively. In GSC20 and GSC267 cells, PDIA3P1 knockdown decreased CD44 expression and increased SOX2 expression, which was verified by IF assays (Fig. 4F and Fig. S6E). In addition, two MES marker proteins, CD44 and YKL-40, were downregulated after interfering with PDIA3P1 expression (Fig. 4H).

### PDIA3P1 promotes PMT and TMZ resistance by affecting C/EBPβ in GSCs

**Fig. 5:**
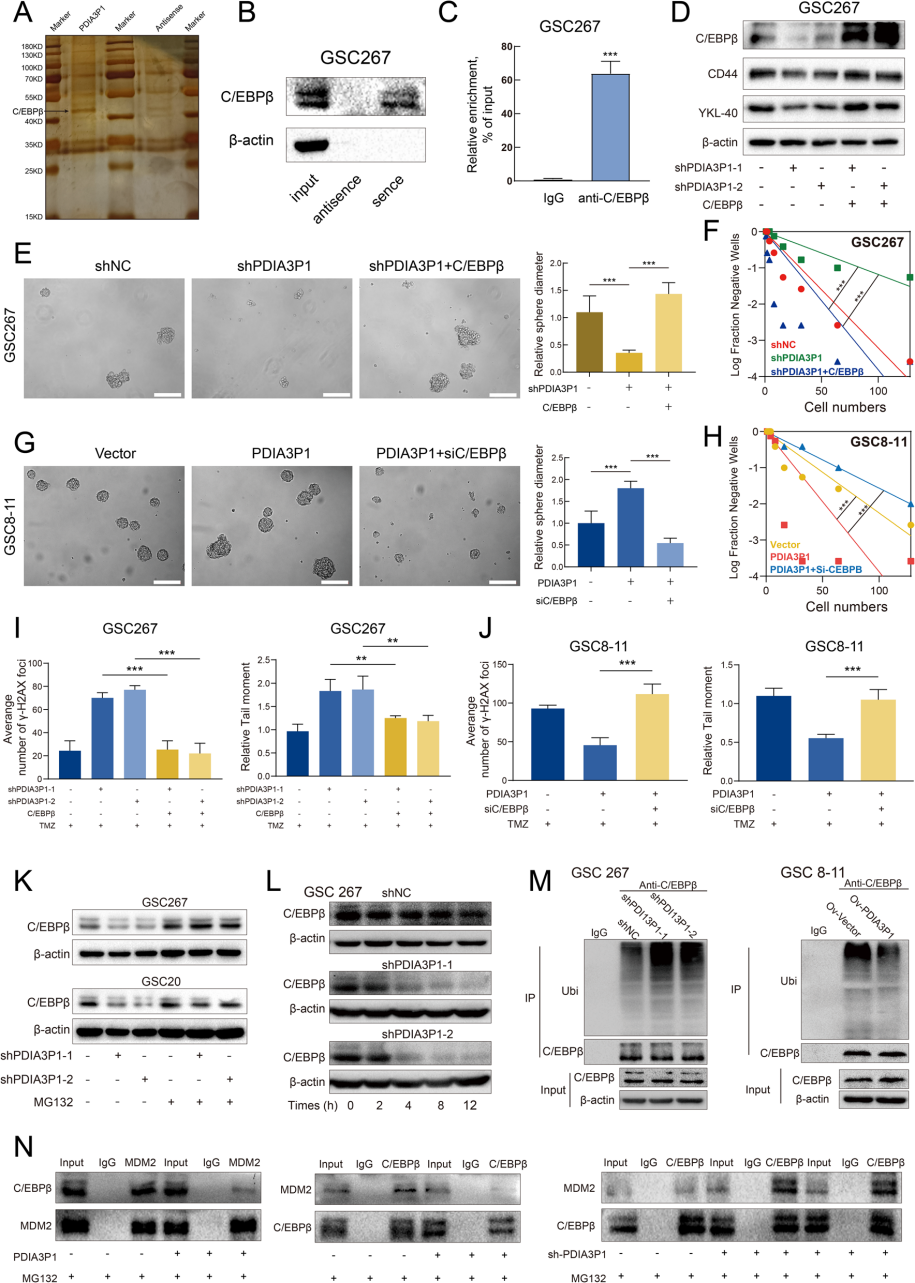
PDIA3P1 stabilizes C/EBPβ by preventing MDM2 -mediated ubiquitination. **A** Different protein bands pulled down by PDIA3P1 junction sense or anti-sense in GSC267 cells. **B** RNA pull down assay showing the interaction between C/EBPβ with PDIA3P1. **C** RIP and qRT-PCR assays revealing the interaction between C/EBPβ with PDIA3P1. **D** The protein expression effected by PDIA3P1 knockdown and C/EBPβ overexpression. **E F** Overexpression of C/EBPβ rescued the effect of PDIA3P1 knockdown on self-renewal ability of GSC267. Scale bar, 200 μm. **G H** Knockdown of C/EBPβ inhibited the effect of PDIA3P1 overexpression on self-renewal ability of GSC8–11. Scale bar, 200 μm. **I** Quantification of comet assay and γ-H2AX staining of GSC267 under TMZ treatment (400 μM, 48 h). **J** Quantification of comet assay and γ-H2AX staining of GSC8–11 under TMZ treatment (400 μM, 48 h). **K** Western blotting analysis of the effect of PDIA3P1 knockdown on C/EBPβ with or without MG132 treatment (10 μM, 12 h). **L** Western blotting analysis of C/EBPβ in PDIA3P1 stable knockdown and control GSC267 cells after treatment with CHX (100 μg/ml) for indicated times. **M** GSCs lysates were immunoprecipitated with anti-C/EBPβ antibody followed by immunoblotting with anti-Ubiquitin antibody and anti-C/EBPβ antibody. The GSCs were pretreated with MG132 (10 μM) for 6 hours. **N** Co-IP analysis of interaction between C/EBPβ and MDM2 in GSC267 cells transfected with PDIA3P1 or shPDIA3P1

Given that lncRNAs can directly bind to proteins to exert regulatory functions, we first performed RNA pull-down experiments in GSC267 cells to explore the molecular interaction mechanism of PDIA3P1. Then, we detected the binding proteins of PDIA3P1 using silver staining and mass spectrometry analysis (Table. S4). We found that C/EBPβ was significantly enriched on PDIA3P1 compared to antisense, and RNA pull down assay was performed again to verify their interaction (Fig. 5A, B). Subsequently, an RNA immunoprecipitation (RIP) assay further confirmed that C/EBPβ specifically combines with PDIA3P1 (Fig. 5C). C/EBPβ is thought to be one of the MRs promoting PMT, and the interaction and effect of PDIA3P1 on C/EBPβ expression further validated our finding that PDIA3P1 is involved in GBM PMT progression. Knockdown of PDIA3P1 decreased the expression of C/EBPβ, CD44 and YKL-40, whereas transfection of C/EBPβ into GSC267 counteracted the effect of PDIA3P1 knockdown on the expression of CD44 and YKL-40 (Fig. 5D). We further performed neurosphere formation assays and ELDA to explore the effect of PDIA3P1- C/EBPβ on tumorigenesis. We observed the expansion of tumorspheres, and the ability to form spheres was significantly restored when C/EBPβ was transfected into PDIA3P1-knockdown GSC267 cells (Fig. 5E, F). Knockdown of C/EBPβ in PDIA3P1-expressing GSC8–11 cells suppressed tumorsphere expansion and reduced sphere formation ability (Fig. 5G, H). To investigate whether PDIA3P1 promotes the resistance of GSCs to TMZ by affecting C/EBPβ, the comet assay and γ-H2AX IF assay were performed. The results showed that knockdown of PDIA3P1 increased nuclear γ-H2AX levels in GSC267 in response to TMZ treatment, whereas transfection of C/EBPβ into shPDIA3P1-GSC267 decreased nuclear γ-H2AX expression, implying that overexpression of C/EBPβ restores TMZ resistance of GSCs (Fig. 5I and Fig. S7A). Similar results were obtained for the comet assay (Fig. 5I and Fig. S7B), suggesting that overexpressing C/EBPβ counteracts the effect of PDIA3P1 knockdown on TMZ resistance. For GSC8–11, the comet assay and γ-H2AX IF assay revealed that knockdown of C/EBPβ restored the sensitivity of GSC8–11 cells expressing PDIA3P1 to TMZ (Fig. 5J, Fig. S7C, D). Collectively, these results determined that PDIA3P1 promotes PMT and TMZ resistance by affecting C/EBPβ expression.

### PDIA3P1 stabilizes C/EBPβ by preventing MDM2-mediated ubiquitination

We further investigated the interaction of PDIA3P1-C/EBPβ. PDIA3P1 knockdown decreased protein expression of C/EBPβ (Fig. 5D, K), but not mRNA levels of C/EBPβ (Fig. S7E), suggesting that PDIA3P1 regulates protein levels of C/EBPβ by affecting translational or posttranslational modification. The ubiquitin–proteasome system (UPS) is the primary pathway of protein degradation, and it participates in the degradation of more than 80% of proteins in cells [34]. To confirm the possibility that PDIA3P1 regulates C/EBPβ through the proteasome, GSCs were treated with the proteasome inhibitor MG132. Knockdown of PDIA3P1 significantly decreased the expression of C/EBPβ, whereas MG132-treated GSCs with silenced PDIA3P1 displayed minimal changes in C/EBPβ levels (Fig. 5K). Then, we blocked protein synthesis using cycloheximide (CHX) and found that PDIA3P1 knockdown significantly shortened the half-life of C/EBPβ protein in GSC267 cells (Fig. 5L). Consistently, the half-life of the C/EBPβ protein in GSC8–11 cells stably overexpressing PDIA3P1 was significantly longer than that in the corresponding control cells (Fig. S7F). Immunoprecipitation (IP) results demonstrated that knockdown of PDIA3P1 significantly increased the ubiquitylation of C/EBPβ in GSC267 cells, whereas overexpression of PDIA3P1 significantly decreased the ubiquitylation of C/EBPβ in GSC8–11 cells (Fig. 5M). Taken together, our data suggest that PDIA3P1 is involved in the posttranslational modification of C/EBPβ.

E3 ubiquitin ligases are a family of more than 700 proteins that bind ubiquitin to target proteins and play a major role in protein degradation [34]. To further investigate the E3 ubiquitin ligases involved in the posttranslational modification of C/EBPβ, we reviewed numerous references and found that mouse double minute 2 homolog (MDM2) targets C/EBPβ for degradation [35]. MDM2 is an E3 ubiquitin ligase of the RING finger family that is involved in the degradation of p53 [36]. Since we confirmed that PDIA3P1 directly binds to C/EBPβ to affect the ubiquitination levels of C/EBPβ, we hypothesized that PDIA3P1 may impact C/EBPβ-MDM2 complex formation. To test this hypothesis, the interaction between C/EBPβ and MDM2 was investigated using co-IP assays. The results demonstrated that overexpression of PDIA3P1 hampered the interaction between C/EBPβ and MDM2 in GSC267 cells. In addition, knockdown of PDIA3P1 resulted in increased MDM2 binding to C/EBPβ in GSC267 cells (Fig. 5N). Collectively, our data suggest that PDIA3P1 stabilizes C/EBPβ by disrupting the C/EBPβ-MDM2 complex.

### PDIA3P1 is upregulated in response to TMZ-induced activation of the p38-MAPK signaling pathway

**Fig. 6:**
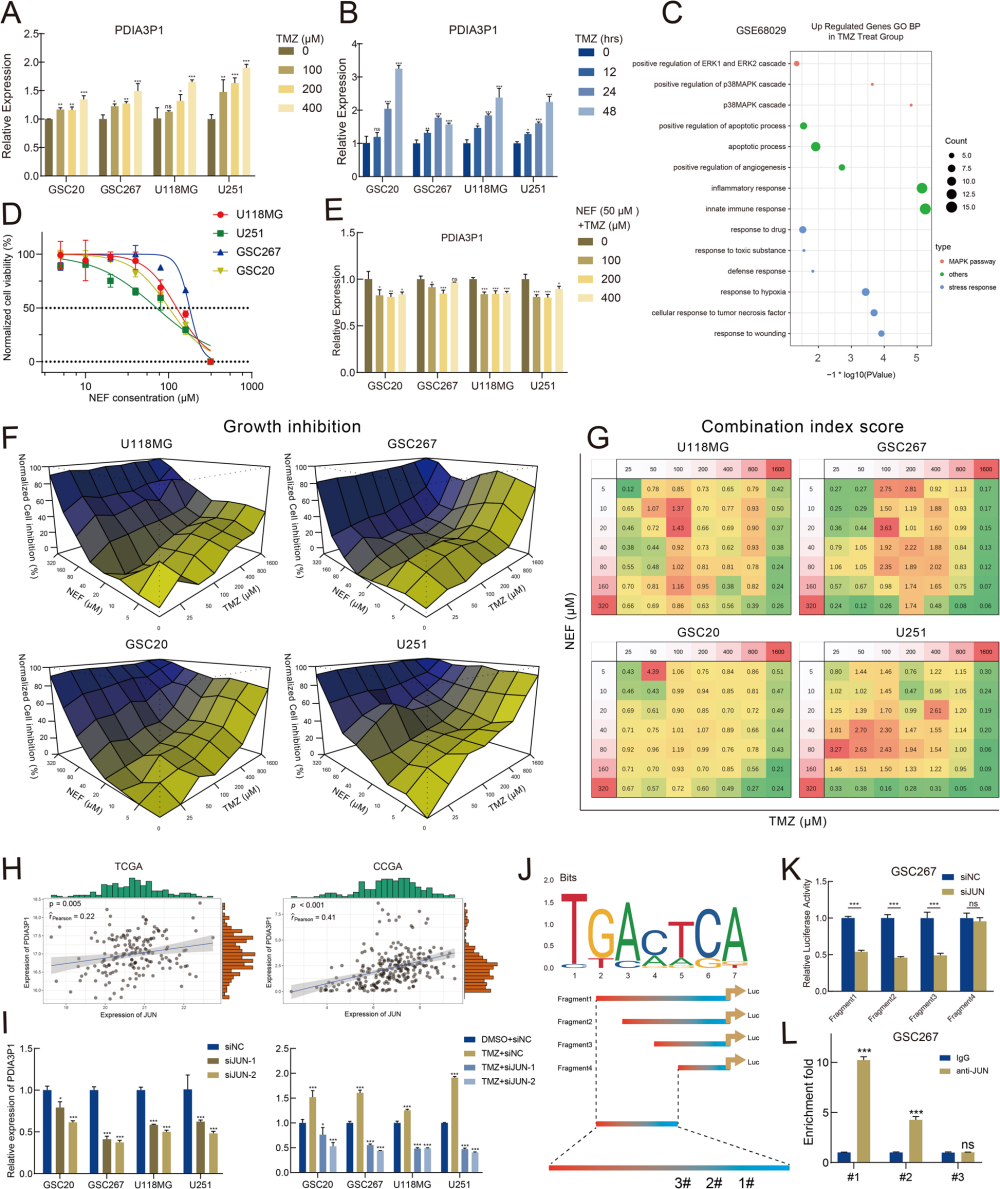
PDIA3P1 is upregulated in response to TMZ-induced activation of the p38-MAPK signaling pathway. **A B** PDIA3P1 expression was induced by TMZ treatment in a dose-dependent (treatment with different concentrations of TMZ for 48 hours) and time-dependent (treatment with 400 μM TMZ for indicated times) manner. **C** Bubble plot visualized the significantly enriched GO biological pathways using genes upregulated in TMZ-treated GSC group in GSC68029. **D** Cell viability assay in GSC20, GSC267, U118MG, and U251 treated with different concentrations of NEF for 48 h. **E** NEF treatment (50 μM, 48 h) abrogated elevation of PDIA3P1 expression induced by TMZ treatment. **F** Cells were treated with TMZ in combination with NEF at different concentrations and percentages of growth inhibition were visualized. **G** CI scores of cells treated with TMZ in combination with NEF at different concentrations. **H** Pearson correlation test was performed to show the correlation of PDIA3P1 expression with JUN in TCGA and CGGA datasets, respectively. **I** Knocking down of JUN expression using siRNA reduced the expression of PDIA3P1 (left panel). Knocking down of JUN counteracted TMZ treatment (400 μM, 48 h) induced upregulation of PDIA3P1 (right panel). **J** The recognition motif of JUN obtained from the JASPAR (upper panel) and schematic illustration of four fragments in promoter sequence of PDIA3P1 (lower panel). **K** The luciferase assay showed PDIA3P1 knockdown reduced promoter activity in fragments 1–3. **L** ChIP-PCR assay showed that JUN bound to a predicted site within the PDIA3P1 promoter

TMZ treatment and subsequent detrimental stress within tumor cells can change the expression levels of multiple genes. We treated GSC20, GSC267, U118MG and U251 cells with different concentrations of TMZ for 48 h or with 400 μM TMZ for different durations. We observed that PDIA3P1 expression was upregulated in a dose- and time-dependent manner following TMZ intervention (Fig. 6A, B). To explore the mechanism of TMZ-induced PDIA3P1 upregulation, we obtained RNA array data from GSE68029, which identified defense profiles of GSCs in response to 500 μM TMZ. We performed differential analysis of these data and conducted gene oncology (GO) enrichment analysis on the differentially expressed genes. Compared to parental GSCs, TMZ-resistant GSCs were significantly enriched in gene sets associated with the p38α MAPK biological pathway, suggesting that the p38α MAPK signaling pathway could be involved in TMZ resistance and is activated in response to TMZ treatment of GSCs (Fig. 6C). Additionally, the expression of phospho-P38 were upregulated with increasing concentration of TMZ treatment, while there was a slight down-regulation of P38 expression following TMZ treatment (Fig. S7G). The p38α MAPK signaling pathway is primarily responsible for the transduction of extracellular signals, which can be activated by various environmental stressors and inflammatory cytokines [37]. Activation of the core molecule p38α indirectly regulates the transcriptional process of various genes by regulating multiple transcription factors, helping cells respond adequately to changing environmental conditions [38].

Targeting p38α may block the stress response of tumor cells, preventing TMZ-induced upregulation of PDIA3P1. Therefore, we reviewed small molecule inhibitors specifically targeting p38 from DRUGBANK and MCE. We screened NEF as a potential drug for the treatment of Alzheimer’s disease (AD), which has been preliminarily confirmed to be safe for human use [39]. NEF has excellent BBB permeability, suggesting its value for CNS disorders, and some studies have demonstrated antitumor activity of NEF [40–42]. CCK-8 cell proliferation was performed to determine the IC_50_ of NEF in four cell lines (Fig. 6D). GBM cells treated with NEF inhibited TMZ-induced upregulation of PDIA3P1 (Fig. 6E). Then, we further explored whether TMZ combined with NEF could synergistically inhibit GBM cell growth. GBM cells were treated with the indicated concentrations of TMZ and NEF, and cell growth inhibition was assessed using the CCK-8 assay (Fig. 6F). Based on the results of proliferation inhibition, we calculated the combination index (CI) score to evaluate the combined effect of TMZ and NEF (Fig. 6G and Fig. S8A). CI > 1.25, CI = 0.75–1.25, and CI < 0.75 were defined as antagonistic, additive and synergistic effects, respectively. For instance, in GSC267 cells, a relatively low concentration of TMZ (50 μM) and NEF (20 μM) may exhibit a better synergistic effect (CI = 0.44), despite their relatively low growth inhibitory effects of approximately 23%. When GSC20 cells were treated with moderate concentrations of TMZ (800 μM) and NEF (80 μM), they exhibited an additive effect despite their relatively high growth inhibition of approximately 81%. Collectively, these data reveal that TMZ in combination with NEF exhibits synergistic effects at the indicated concentrations.

Activation of the p38-MAPK signaling pathway could further activate certain transcription factors, such as JUN. We observed a significantly positive relationship between expression of JUN and PDIA3P1 in the TCGA and CGGA datasets (Fig. 6H). Knockdown of JUN not only reduced PDIA3P1 expression but also counteracted TMZ-induced upregulation of PDIA3P1 (Fig. 6I), preliminarily suggesting that JUN is responsible for PDIA3P1 transcription. We constructed four fragments of different lengths located upstream of the TSS based on the JUN binding motif (Fig. 6J). The four luciferase reporter plasmids were transfected into GSC267 cells individually to verify their JUN binding sites. The luciferase activity of fragment 4 was statistically unchanged after knockdown of JUN, demonstrating that JUN does not bind to fragment 4 (Fig. 6K). To further determine the binding sites in more detail, we designed three pairs of PCR primers and performed a ChIP assay. The qRT–PCR assay yielded approximately 10-fold enrichment and 5-fold enrichment for site #1 and site #2, respectively, while there was no significant enrichment at site #3 (Fig. 6L and Fig. S8B). In conclusion, our data suggest that JUN is involved in TMZ-induced upregulation of PDIA3P1 and directly binds and initiates PDIA3P1 transcription.

### NEF combined with TMZ confers a better antitumor effect both in vitro and in vivo

**Fig. 7:**
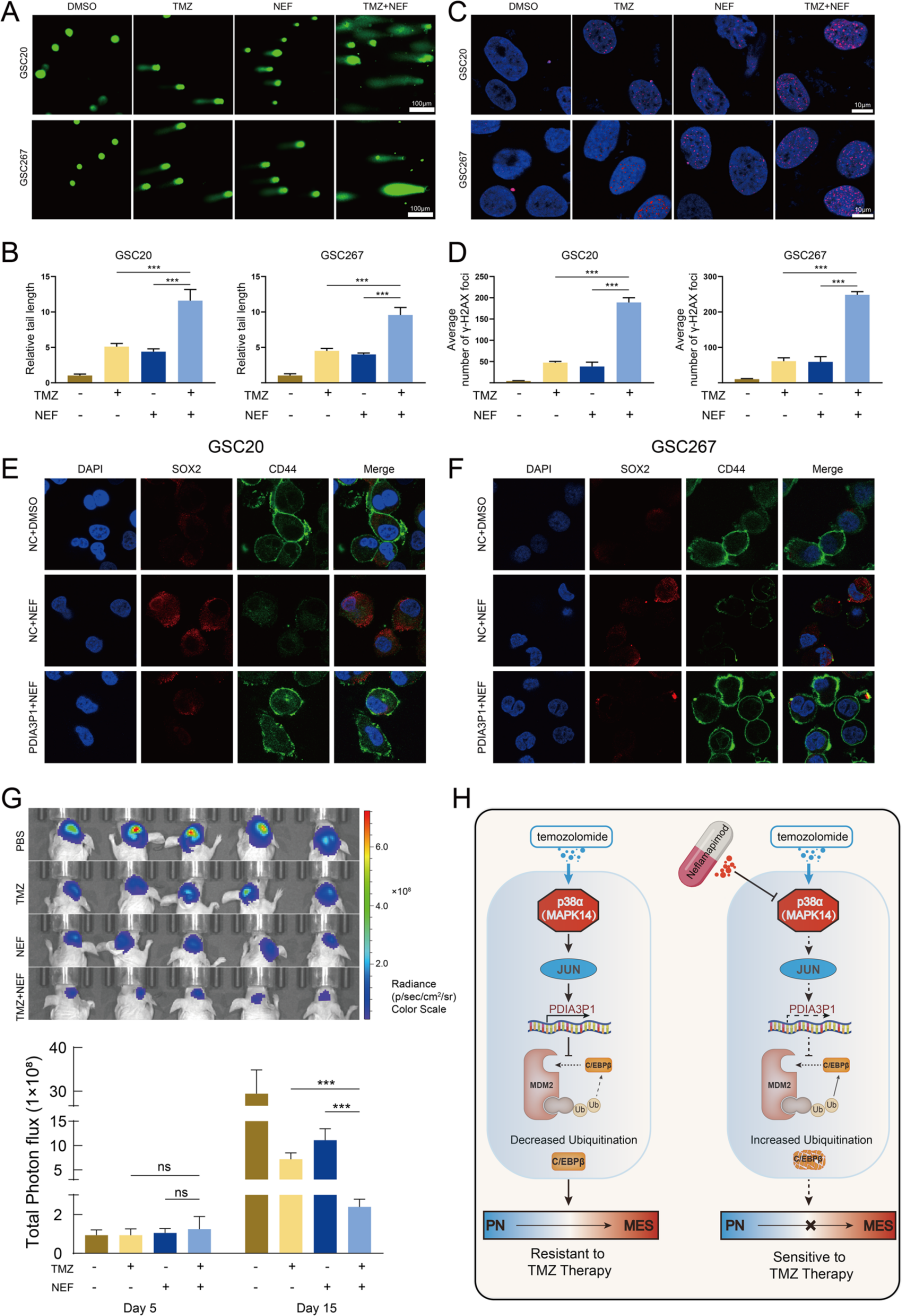
NEF combined with TMZ confers a better anti-tumor effect both in vitro and in vivo. **A B** The representative images (**A**) and quantification (**B**) of comet assay showed that TMZ (400 μM, 48 h) combined with NEF (50 μM, 48 h) contributed a stronger DNA damage effect in GSC20 and GSC267, respectively. Scale bar, 100 μm. **C D** The representative images (**C**) and quantification (**D**) of γ-H2AX staining in GSC20 and GSC267 (TMZ 400 μM, 48 h. NEF 50 μM, 48 h). Scale bar, 10 μm. **E F** Representative images of IF staining showed the effect of NEF treatment (50 μM, 48 h) and PDIA3P1 overexpression on the expression of CD44 and SOX2 in GSC20 (**E**) and GSC267 (**F**), respectively. Scale bar, 10 μm. **G** Bioluminescence imaging (upper panel) and quantification (lower panel) of tumor size in GSC267 xenografted nude mice treated with PBS, NEF (5 mg/kg, p.o., 5 days per week), TMZ (5 mg/kg, p.o., 5 days per week) or both drugs in combination. **H** Working model plot showing that PDIA3P1 plays a key role in promoting the TMZ resistance of GBM cells. The p38α-JUN was activated by TMZ treatment and promoting the transcription of PDIA3P1. PDIA3P1 disrupted the MDM2-C/EBPβ complex to stabilize C/EBPβ and promote PMT, thereby promoting the resistance of GBM cells to TMZ treatment. NEF, a highly selective p38α inhibitor, inhibited TMZ-induced upregulation of PDIA3P1 expression and provided a promising strategy to address the challenge of TMZ resistance in glioma cells

To evaluate the antitumor effect of the TMZ and NEF combination, we conducted a series of in vitro experiments. In GSC20 and GSC267 cells, the comet assay showed that levels of DNA damage were higher when treated with TMZ or NEF alone than in the control group, while DNA damage was more pronounced in the combination treatment group than in either monotherapy group, indicating that the combination of TMZ and NEF exhibits a more powerful antitumor effect (Fig. 7A, B). Similar results were obtained in the ɣH2AX IF assay, where significantly higher nuclear ɣH2AX was observed in the TMZ and NEF combined group, suggesting that combined treatment resulted in a potentially enhanced DNA damage effect (Fig. 7C, D). The EdU assay showed that either TMZ or NEF treatment alone inhibited the proliferation of tumor cells, whereas the combination group exhibited a more obvious inhibition of proliferation efficiency (Fig. S9A). Next, we detected apoptosis levels using flow cytometry. U118MG cells treated with TMZ or NEF alone exhibited 31.35 and 30.22% apoptosis rates, respectively, whereas the apoptosis rate increased to 51.2% when TMZ was combined with NEF (Fig. S9B). Given that NEF targets p38α and thereby affects the subsequent transcriptional process of PDIA3P1, which has been shown to promote PMT, we next examined whether NEF is also involved in the subtype of GSC. The results showed that expression of CD44 was significantly reduced in GSC20 and GSC267 cells after 48 hours of NEF (50 μM) treatment, while expression of SOX2 was elevated. In addition, overexpression of PDIA3P1 counteracted the effect of NEF treatment on CD44 and SOX2 expression, indicating that NEF affects the subtypes of GSCs through PDIA3P1 (Fig. 7E, F). To evaluate the antitumor activities of TMZ and/or NEF in vivo, nude mice carrying GSC267 xenografted tumors were administered TMZ (5 mg/kg, p.o., 5 days per week), NEF (5 mg/kg/day, p.o., 5 days per week), or both drugs in combination after inducing an orthotopic xenograft model. The results showed that either TMZ or NEF treatment alone inhibited the proliferation of tumor cells, whereas the combination treatment produced remarkable tumor regression (Fig. 7G and Fig. S9C). Consistently, survival analysis showed that either TMZ or NEF treatment alone prolonged the survival time of mice, whereas the combination treatment group displayed a significantly longer survival time (Fig. S9D). H&E-stained mouse brain sections showed that TMZ combined with NEF limited the invasion ability of the tumor to the greatest extent (Fig. S9E). Taken together, our results demonstrated that TMZ in combination with NEF exerts excellent synergistic antitumor effects both in vitro and in vivo.

## Discussion

In this study, we screened the lncRNA PDIA3P1, which is closely related to TMZ resistance in GBM, based on a comprehensive analysis of the CCLE and GDSC databases. Bioinformatics analyses of public databases combining qRT–PCR results indicated that expression of PDIA3P1 was upregulated in TMZ-resistant cell lines and predicted a higher risk of tumor recurrence. Combining in vitro and in vivo assays, we further confirmed that PDIA3P1 reduces the TMZ sensitivity of glioma cell lines. Mechanistically, PDIA3P1 promoted PMT by disrupting the C/EBPβ/MDM2 complex to inhibit the ubiquitination of C/EBPβ, enabling glioma cells to obtain stronger TMZ therapy resistance. To our knowledge, this is the first report showing the function and mechanism of PDIA3P1 in promoting TMZ resistance in GBM.

The primary obstacle to GBM therapy is the development of TMZ resistance. Increasing evidence suggests that excessive activation of O6-methylguanine-DNA methyltransferase (MGMT), which removes TMZ-induced alkylation from different nucleotides, is the most important cause of TMZ resistance in GBM [43–45]. However, studies have recently shown that MGMT overexpression is not the only determinant contributing to GBM resistance to TMZ [5]. Other factors, such as the advent of GSCs, overactivation of DNA repair pathways, favorable autophagy, decreased drug influx and increased drug efflux, facilitate drug resistance in TMZ in addition to MGMT overexpression [46–51]. GSCs exhibit the capacity for self-renewal, immortal propagation and multilineage differentiation [52]. GSCs can be divided into PN and MES subtypes according to their transcriptional program, genotype and epigenetic status [19, 53, 54]. PN GSC is characterized by relatively faster proliferation and sensitivity to adverse stimulation, whereas MES GSC is characterized by the secretion of various factors and the ability to maintain relative stability under adverse conditions [33]. The PN subtype transition to the MES subtype is considered a crucial process for tumor recurrence and treatment tolerance in GBM [55]. It was reported that immune infiltration in the TME is associated with PMT. However, our analysis indicated that expression of PDIA3P1 was not associated with tumor immunity (Fig. S3B and Table. S3), suggesting the impact of PDIA3P1 on PMT based on an endogenous pathway.

C/EBPβ is highly expressed and activated in MES subtype GSCs and is the MR in the process of PMT. Given its role in the PMT, C/EBPβ has great potential as a therapeutic target for GBM [26]. However, the mechanisms for C/EBPβ regulation in GBM have not been completely clarified. Based on RNA pulldown and mass spectrometry analysis, we concluded that PDIA3P1 promotes PMT by targeting C/EBPβ. We found that PDIA3P1 had no effect on mRNA expression but did increase C/EBPβ protein expression in GSCs by increasing C/EBPβ protein stability and decreasing C/EBPβ ubiquitination. Therefore, our results suggest that PDIA3P1 functions as a regulator of PMT by restricting C/EBPβ degradation. PDIA3P1 has been postulated to primarily function as a competitive endogenous RNA (ceRNA) that competes for microRNA (miRNA) binding, playing an important role in gene regulation [56–58]. In this study, PDIA3P1 did not function as a ceRNA but was able to physically bind to C/EBPβ protein, reducing its ubiquitination and subsequent degradation. Ubiquitin-dependent protein degradation plays a critical role in the posttranscriptional regulation of most proteins [59]. It has been reported that C/EBPβ can be degraded by the E3 ubiquitin ligase MDM2 to promote myogenesis [60]. Therefore, we hypothesized and verified that PDIA3P1 affects the ubiquitination and degradation of C/EBPβ through MDM2. Our data suggest that PDIA3P1 binds proteins that function to disrupt the C/EBPβ/MDM2 complex rather than binding to miRNAs.

The function of the p38α-MAPK pathway is to relay, amplify and integrate a variety of extracellular stresses, such as radiotherapy, chemotherapy, hypoxia and hunger, thereby regulating the genomic and physiological response of cells to their environment [61]. It has been reported that acute treatment with TMZ induces DNA damage and transitory activation of MAPK14/p38α [62, 63]. In addition, activation of the MAPK pathway has been associated with poor survival in GBM patients during the TMZ era [64]. The p38α-MAPK pathway is markedly activated during TMZ treatment and resists the killing effect of TMZ. We found that expression of PDIA3P1 increased after treatment of cells with TMZ in a concentration- and time-dependent manner. Further analyses indicated that the p38α-MAPK signaling pathway mediated TMZ-induced upregulation of PDIA3P1. There is a loop in which TMZ treatment activates the p38α-MAPK signaling pathway, which then promotes the expression of PDIA3P1, finally resulting in PDIA3P1 promoting PMT to attenuate the adverse effects of TMZ treatment. We next aim to test interventions to break this loop and provide potential therapeutic strategies for overcoming TMZ resistance.

Currently, TMZ combined with other antitumor agents has become the primary strategy for treating refractory glioma [65]. The basic principle of combination treatment is to leverage different agents that target key pathways by different mechanisms to reduce drug-resistant cancer cells. There is evidence that combination therapy with TMZ prolongs the overall survival of GBM patients [66]. Based on the strategy of combination therapeutics, we selected a specific p38α inhibitor, NEF, which exhibits BBB permeability. We revealed that NEF in combination with TMZ exhibits synergistic effects at the indicated concentrations. Moreover, we confirmed the efficacy of the combined treatment strategy using both in vitro and in vivo experiments. In summary, we determined the mechanism by which PDIA3P1 mediates TMZ resistance. More importantly, we demonstrated a new treatment strategy in which the combined use of TMZ and NEF has the potential to overcome TMZ resistance.

## Conclusions

In conclusion, PDIA3P1 promotes PMT through stabilization of C/EBPβ, conferring GBM cell resistance to TMZ. P38α-JUN is responsible for the transcriptional upregulation of PDIA3P1 induced by TMZ intervention. The p38α-targeted drug NEF prevents TMZ-induced upregulation of PDIA3P1. NEF combined with TMZ exhibits excellent synergistic antitumor effects (Fig. 7H). Our research provides a clinical translational basis for the possibility of overcoming TMZ resistance and recurrence of GBM.

## Supplementary Information

**Additional file 1: Supplementary Figure 1. A** The IC50 for TMZ and expression of PDIA3P1 of 10 glioma cell lines (TMZ sensitive and TMZ resistant cell lines). **B** Patients with high PDIA3P1 expression exhibited shorten progress disease survival time. **C** The PDIA3P1 expression in 4 glioblastoma cell lines. **D** PDIA31P was knocked down in U118MG and U87MG cells by two different shRNA and overexpressed in LN229 and U251 cells. **E** Cell proliferation was assessed by CCK-8 assay. **Supplementary Figure 2. A B** Bioluminescence imaging of tumor growth on day five in U118MG (**A**) and U251 (**B**) xenograft nude mice. **C D** Kaplan–Meier visualized survival time for animals in different groups for U118MG (**C**) and U251 (**D**). **E F** Representative images of hematoxylin and eosin (H&E) staining in sections from U118MG (**E**) and U251 (**F**) xenografts. Scale bar, 400 μm. **Supplementary Figure 3. A** Patients were divided into high and low PDIA3P1 expression groups according to PDIA3P1 expression, and GSVA analysis was performed, and the results were presented using heatmap. **B** Heatmap visualized immune infiltration using ssGSEA, and the correlation between PDIA3P1 expression and immune infiltration was analyzed using the chi-square test (lower panel). **C** Waterfall plot showing tumor somatic cell mutations in low (left panel) and high (right panel) PDIA3P1 expression groups. **Supplementary Figure 4. A B** DNA damage was assessed by comet (**A**. Scale bar, 100 μm) and γ-H2AX IF staining (**B**. Scale bar, 10 μm) assays. Knockdown of PDIA3P1 significantly promoted TMZ treatment-induced DNA damage. **C D** Cell proliferation was assessed by EdU (**C**. Scale bar, 200 μm) and colony formation (**D**. Scale bar, 200 μm) assays. Knockdown of PDIA3P1 further significantly increased the proliferation inhibitory effect caused by TMZ. The lower panel exhibited the quantification of EdU and colony formation assays. **E F** Representative images of comet (**E**. Scale bar, 100 μm) and γ-H2AX IF staining (**F**. Scale bar, 10 μm) assays for LN229 cells. Overexpression of PDIA3P1 remarkedly reduced TMZ treatment-induced DNA damage. **Supplementary Figure 5. A B** DNA damage was assessed by γ-H2AX IF staining assay in GSC20. Knockdown of PDIA3P1 significantly promoted TMZ treatment-induced DNA damage, whereas overexpression of PDIA3P1 counteracted the DNA damage induced by TMZ treatment (Scale bar, 10 μm). **C D** DNA damage was assessed by comet assay in GSC20 (Scale bar, 100 μm). **E F** Cell proliferation was assessed by EdU assay in GSC20. Knockdown of PDIA3P1 promoted proliferation inhibition caused by TMZ treatment, whereas overexpression of PDIA3P1 partially counteracted TMZ-mediated cell growth inhibition (Scale bar, 200 μm). **Supplementary Figure 6. A** The expression of PDIA3P1 in classical, proneural (PN) and mesenchymal (MES) tissues in the CCGA dataset. **B** ROC curves of PDIA3P1 for MES-GBM subtype prediction in CCGA. **C** Knockdown of PDIA3P1 in GSC20 and GSC267, overexpression of PDIA3P1 in GSC8–11. **D** Neurospheres formation assay revealed knockdown of PDIA3P1 reduced self-renewal capacity of GSC20 and GSC267. Scale bar, 200 μm. The right panels were the quantification of sphere diameters. **E** The quantification of IF staining for SOX2 and CD44 in GSC20 (left panel) and GSC267 (right panel). Knockdown of PDIA3P1 resulted downregulation of CD44 and upregulation of SOX2 in GSC20 and GSC267. **Supplementary Figure 7. A B** Representative images of γ-H2AX IF staining (**A**. Scale bar, 10 μm) and comet (**B**. Scale bar, 200 μm) assays revealed DNA damage in GSC267. **C D** Representative images of γ-H2AX IF staining (**C**. Scale bar, 10 μm) and comet (**D**. Scale bar, 200 μm) assays revealed DNA damage in GSC8–11. **E** The expression of C/EBPβ mRNA detected by qPCR. **F** Western blotting analysis of C/EBPβ in GSC8–11 PDIA3P1 stable overexpressed and control cells after treatment with CHX (100 μg/ml) for indicated times. **G** Western blotting analysis of p-P38 and P38 expression with increasing concentrations of TMZ treatment. **Supplementary Figure 8. A** Visualization of the Fa-CI (fraction afected Combination Index) results obtained form CompuSyn for U118MG, GSC20, GSC267, and U251. **B** The detailed sequence of PDIA3P1 promoter (610–1310) and three predicted binding sites for JUN. **Supplementary Figure 9. A B** EdU assay (**A**. Scale bar, 200 μm) and apoptosis assay (**B**) showed that TMZ combined with NEF exhibited excellent anti-tumor cells effects, respectively. **C** Bioluminescence imaging of tumor growth on day five in GSC267 xenograft nude mice. **D** Kaplan–Meier visualized survival time for mice in different treatment groups. **E** Representative images of hematoxylin and eosin (H&E) staining in sections from GSC267 xenografts. Scale bar, 400 μm.

**Additional file 2: Table S1.** The information of DEGs between TMZ resistant cell lines and TMZ sensitive cell lines. **Table S2.** The Results of GSVA. **Table S3.** The Results of ssGSEA for Tumor Immune Infiltration Analysis. **Table S4.** Mass spectrometry result of proteins related to PDIA3P1.

## Data Availability

All the data obtained and/or analyzed during the current study were available from the corresponding authors on reasonable request.

## Abbreviations

GBM: Glioblastoma
TMZ: Temozolomide
BBB: Blood-brain barrier
CNS: Central nervous system
PDIA3P1: Protein disulfide isomerase family A, member 3 pseudogene 1
MRs: Master regulators
C/EBPβ: CCAAT/enhancer binding protein (C/EBP), beta
TME: Tumor microenvironment
TCGA: The Cancer Genome Atlas
CGGA: Chinese Glioma Genome Atlas
IF: Immunofluorescence
NEF: Neflamapimod
MGMT: O6-methylguanine-DNA methyltransferase
