# Supplementary material for: PDIA3P1 promotes Temozolomide resistance in glioblastoma by inhibiting C/EBPβ degradation to facilitate proneural-to-mesenchymal transition
Source: J Exp Clin Cancer Res. 2022 Jul 15;41:223. doi: 10.1186/s13046-022-02431-0 (PMC9284800; doi:10.1186/s13046-022-02431-0)
Supplement: Supplementary file 1 — Additional file 1: Supplementary Figure 1. A The IC50 for TMZ and expression of PDIA3P1 of 10 glioma cell lines (TMZ sensitive and TMZ resistant cell lines). B Patients with high PDIA3P1 expression exhibited shorten progress disease survival time. C The PDIA3P1 expression in 4 glioblastoma cell lines. D PDIA31P was knocked down in U118MG and U87MG cells by two different shRNA and overexpressed in LN229 and U251 cells. E Cell proliferation was assessed by CCK-8 assay. Supplementary Figure 2. A B Bioluminescence imaging of tumor growth on day five in U118MG (A) and U251 (B) xenograft nude mice. C D Kaplan–Meier visualized survival time for animals in different groups for U118MG (C) and U251 (D). E F Representative images of hematoxylin and eosin (H&E) staining in sections from U118MG (E) and U251 (F) xenografts. Scale bar, 400 μm. Supplementary Figure 3. A Patients were divided into high and low PDIA3P1 expression groups according to PDIA3P1 expression, and GSVA analysis was performed, and the results were presented using heatmap. B Heatmap visualized immune infiltration using ssGSEA, and the correlation between PDIA3P1 expression and immune infiltration was analyzed using the chi-square test (lower panel). C Waterfall plot showing tumor somatic cell mutations in low (left panel) and high (right panel) PDIA3P1 expression groups. Supplementary Figure 4. A B DNA damage was assessed by comet (A. Scale bar, 100 μm) and γ-H2AX IF staining (B. Scale bar, 10 μm) assays. Knockdown of PDIA3P1 significantly promoted TMZ treatment-induced DNA damage. C D Cell proliferation was assessed by EdU (C. Scale bar, 200 μm) and colony formation (D. Scale bar, 200 μm) assays. Knockdown of PDIA3P1 further significantly increased the proliferation inhibitory effect caused by TMZ. The lower panel exhibited the quantification of EdU and colony formation assays. E F Representative images of comet (E. Scale bar, 100 μm) and γ-H2AX IF staining (F. Scale bar, 10 μm) assays for LN229 cells. Overex [file 13046_2022_2431_MOESM1_ESM.docx]

**Supplementary Information**


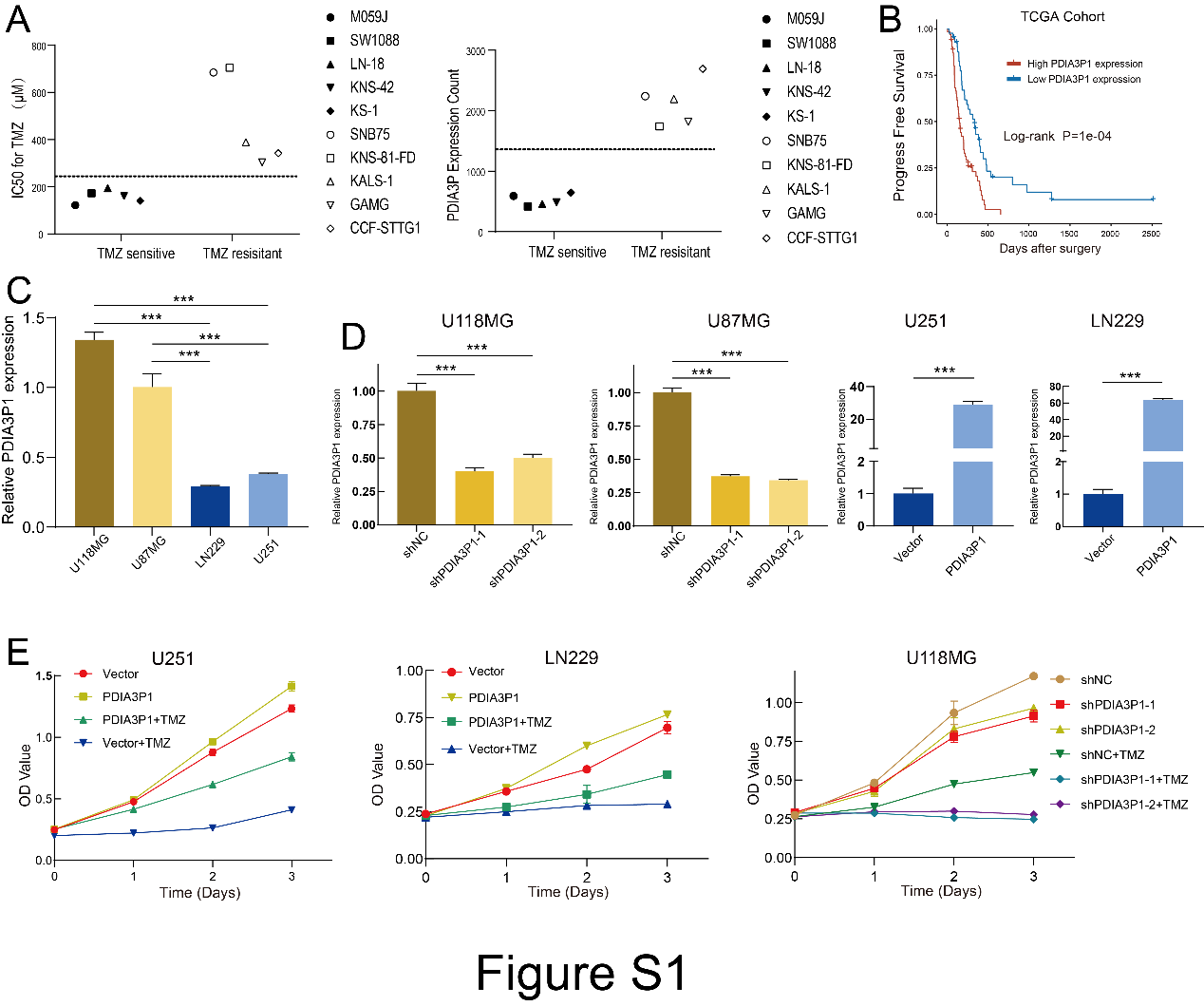


**Supplementary Figure 1**

**A** The IC50 for TMZ and expression of PDIA3P1 of 10 glioma cell lines (TMZ sensitive and TMZ resistant cell lines). **B** Patients with high PDIA3P1 expression exhibited shorten progress disease survival time. **C** The PDIA3P1 expression in 4 glioblastoma cell lines. **D** PDIA31P was knocked down in U118MG and U87MG cells by two different shRNA and overexpressed in LN229 and U251 cells. **E** Cell proliferation was assessed by CCK-8 assay.


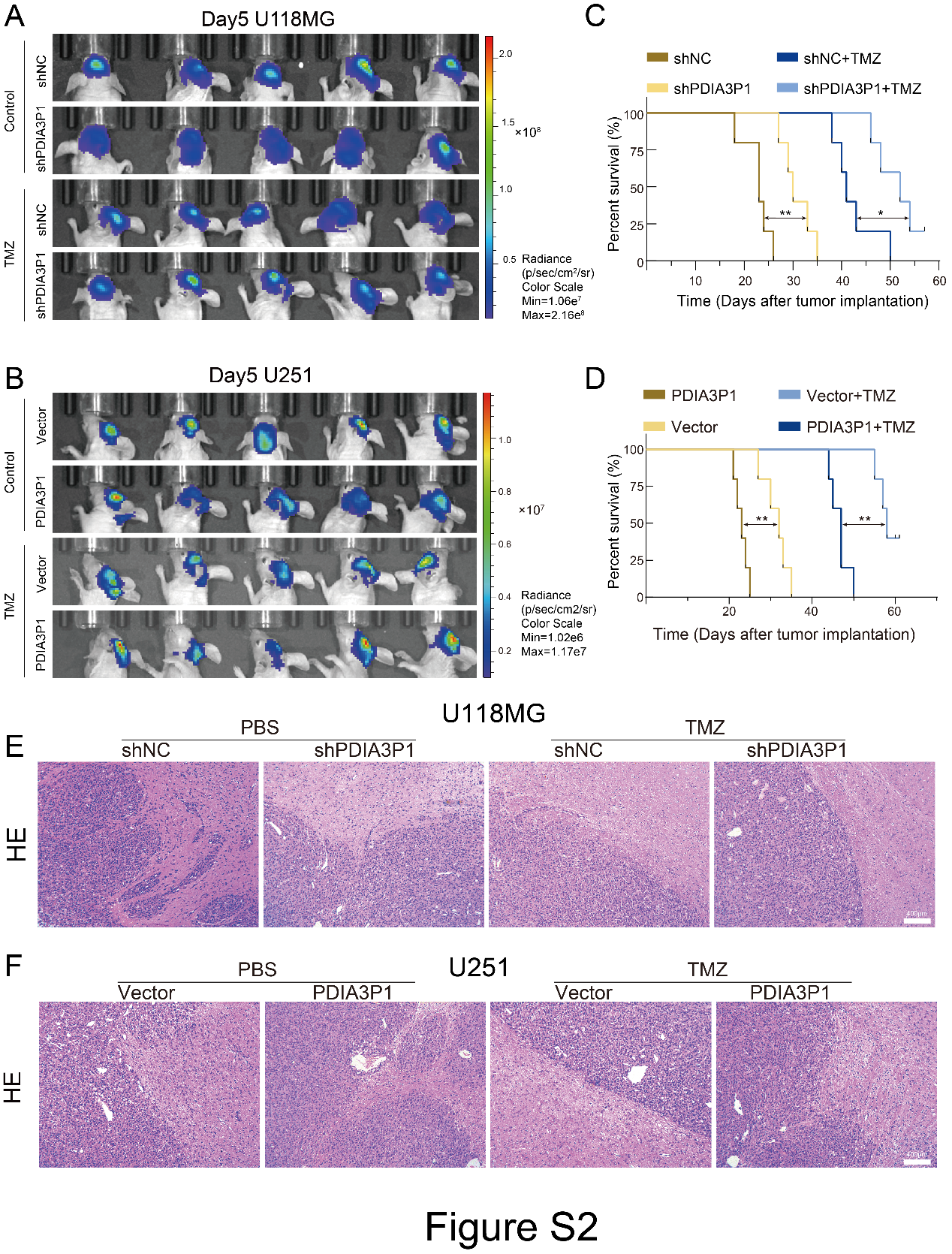


**Supplementary Figure 2**

**A B** Bioluminescence imaging of tumor growth on day five in U118MG (**A**) and U251 (**B**) xenograft nude mice. **C D** Kaplan–Meier visualized survival time for animals in different groups for U118MG (**C**) and U251 (**D**). **E F** Representative images of hematoxylin and eosin (H&E) staining in sections from U118MG (**E**) and U251 (**F**) xenografts. Scale bar, 400μm.


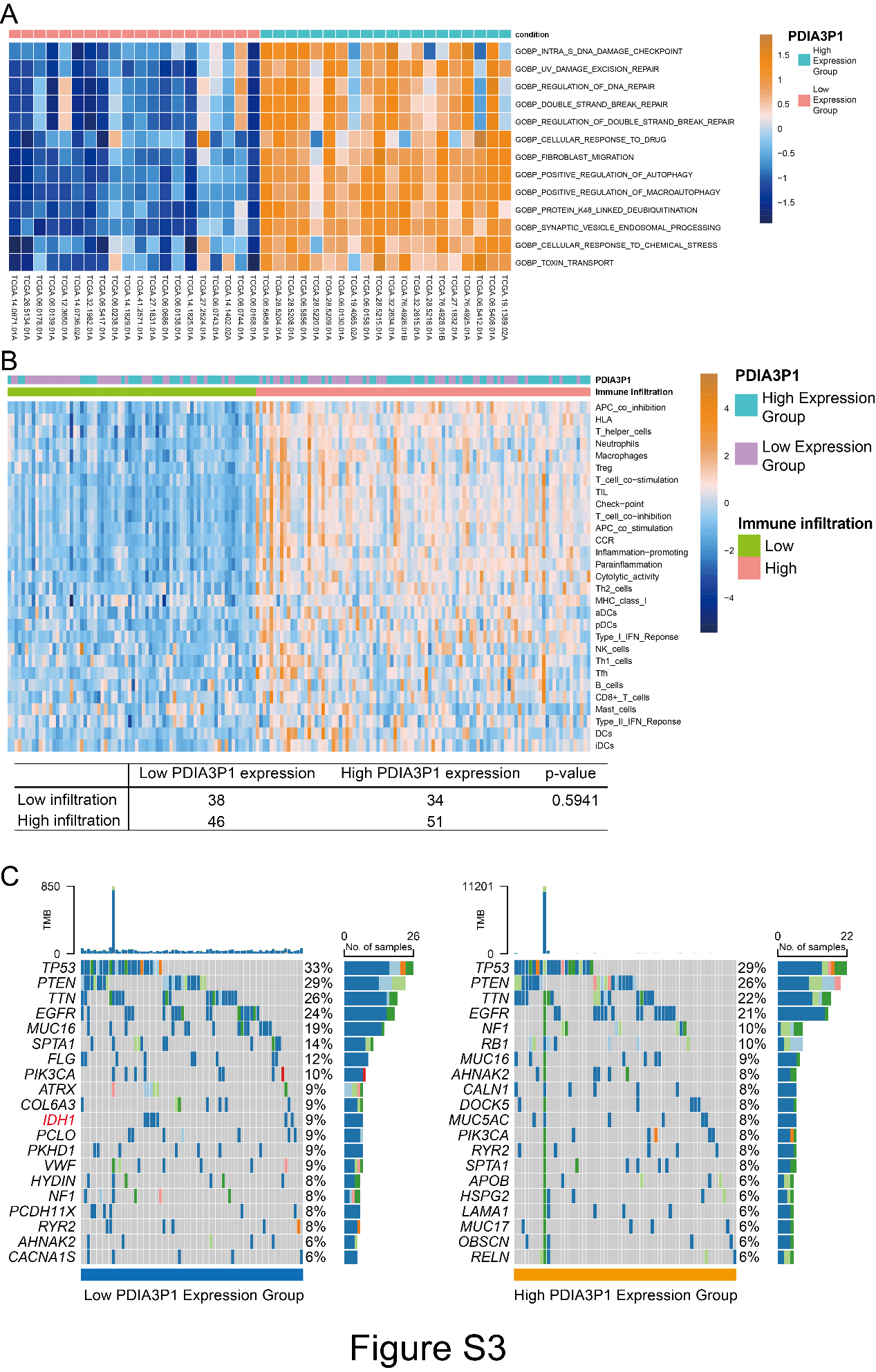


**Supplementary Figure 3**

**A** Patients were divided into high and low PDIA3P1 expression groups according to PDIA3P1 expression, and GSVA analysis was performed, and the results were presented using heatmap. **B** Heatmap visualized immune infiltration using ssGSEA, and the correlation between PDIA3P1 expression and immune infiltration was analyzed using the chi-square test (lower panel). **C** Waterfall plot showing tumor somatic cell mutations in low (left panel) and high (right panel) PDIA3P1 expression groups.


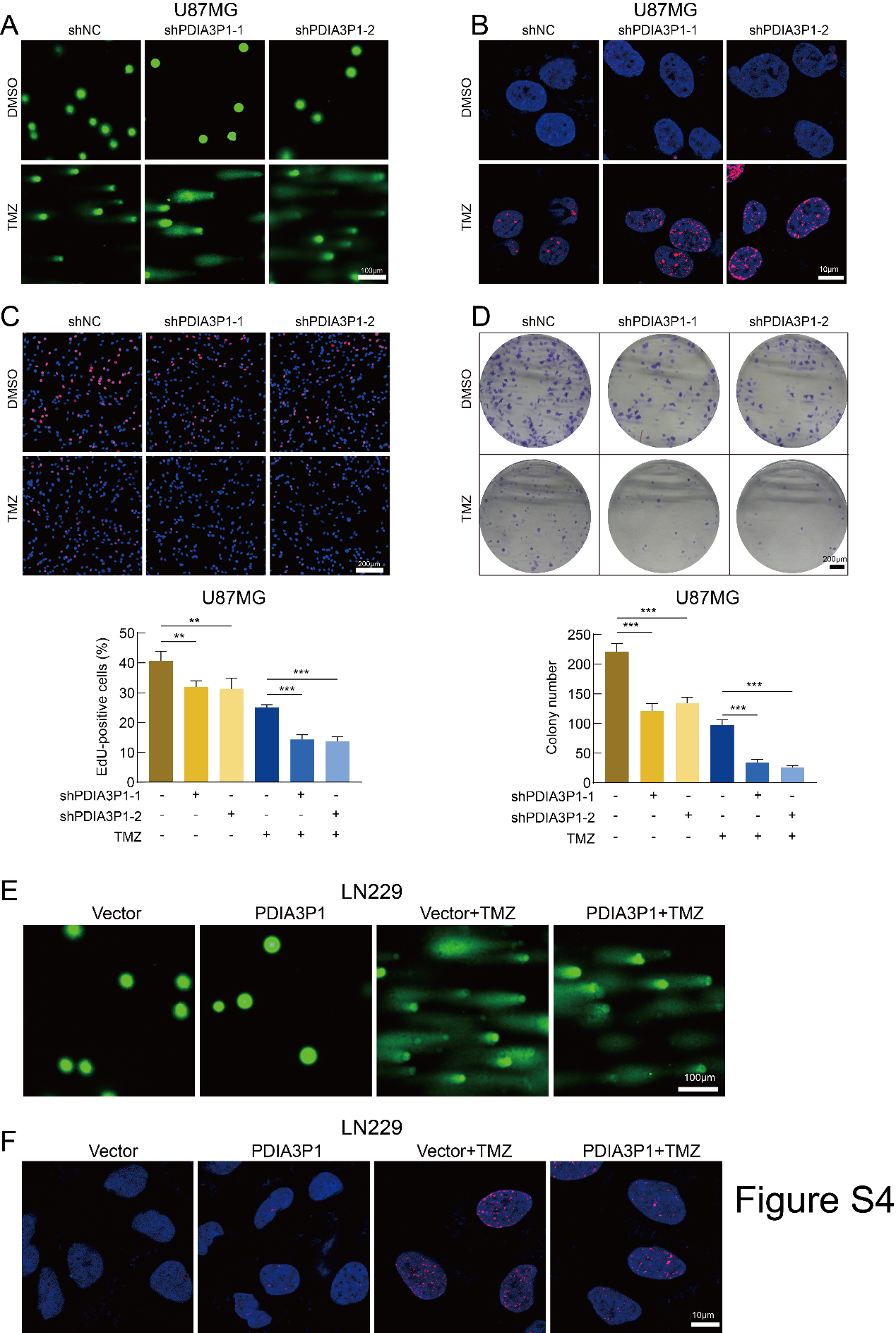


**Supplementary Figure 4**

**A B** DNA damage was assessed by comet (**A**. Scale bar, 100μm) and γ-H2AX IF staining (**B**. Scale bar, 10μm) assays. Knockdown of PDIA3P1 significantly promoted TMZ treatment-induced DNA damage. **C D** Cell proliferation was assessed by EdU (**C**. Scale bar, 200μm) and colony formation (**D**. Scale bar, 200μm) assays. Knockdown of PDIA3P1 further significantly increased the proliferation inhibitory effect caused by TMZ. The lower panel exhibited the quantification of EdU and colony formation assays. **E F** Representative images of comet (**E**. Scale bar, 100μm) and γ-H2AX IF staining (**F**. Scale bar, 10μm) assays for LN229 cells. Overexpression of PDIA3P1 remarkedly reduced TMZ treatment-induced DNA damage.


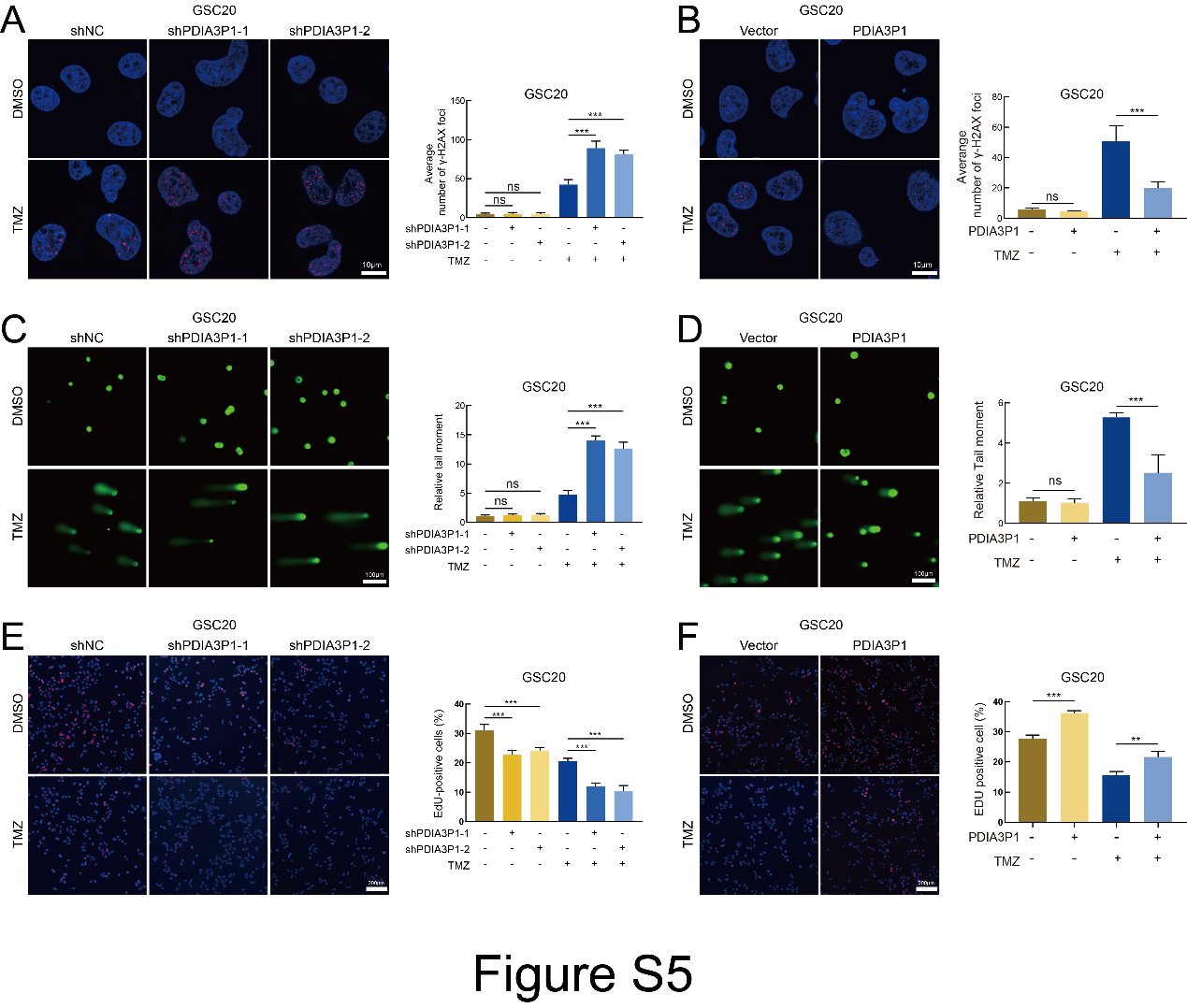


**Supplementary Figure 5**

**A B** DNA damage was assessed by γ-H2AX IF staining assay in GSC20. Knockdown of PDIA3P1 significantly promoted TMZ treatment-induced DNA damage, whereas overexpression of PDIA3P1 counteracted the DNA damage induced by TMZ treatment (Scale bar, 10μm). **C D** DNA damage was assessed by comet assay in GSC20 (Scale bar, 100μm). **E F** Cell proliferation was assessed by EdU assay in GSC20. Knockdown of PDIA3P1 promoted proliferation inhibition caused by TMZ treatment, whereas overexpression of PDIA3P1 partially counteracted TMZ-mediated cell growth inhibition (Scale bar, 200μm).


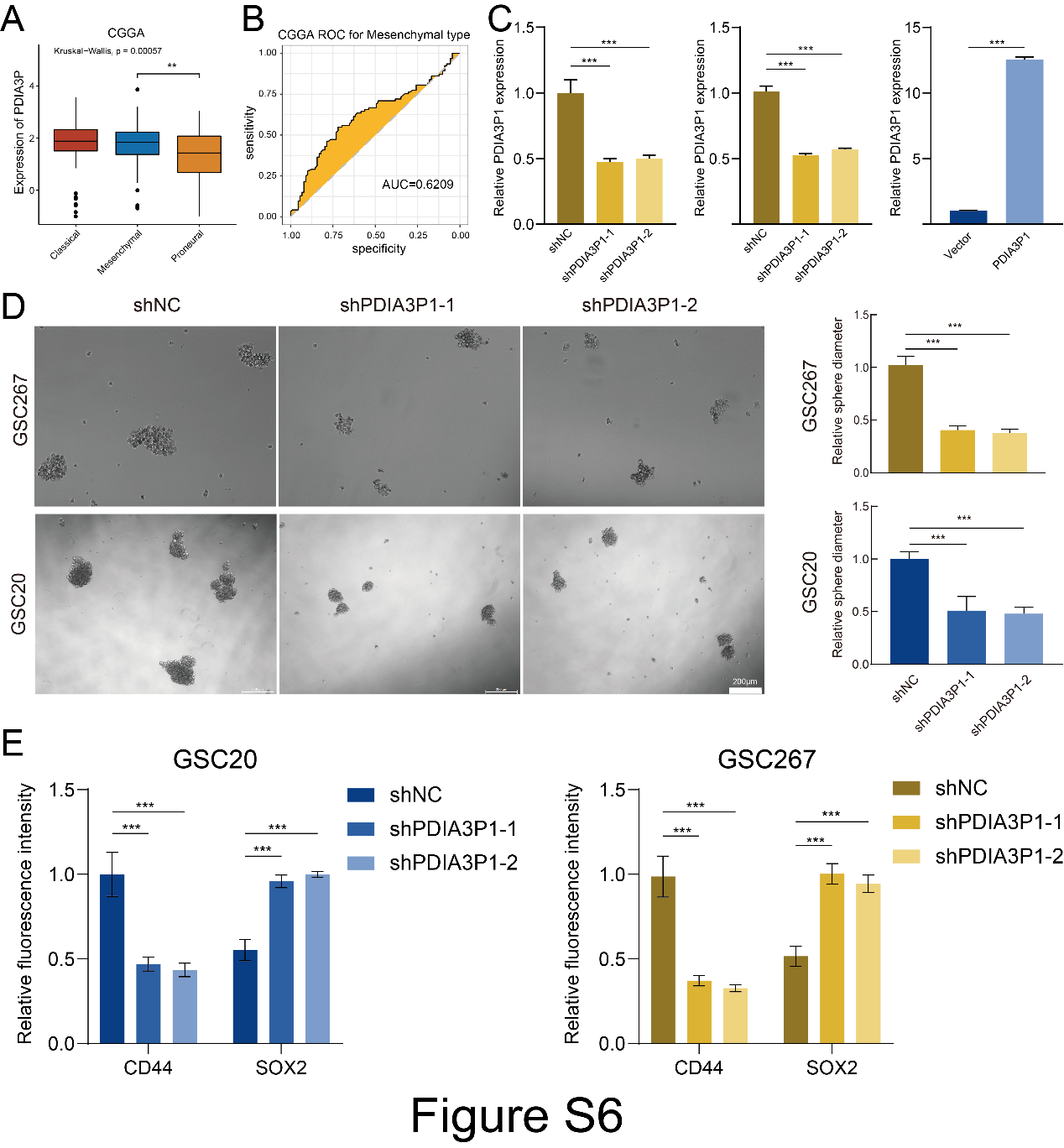


**Supplementary Figure 6**

**A** The expression of PDIA3P1 in classical, proneural (PN) and mesenchymal (MES) tissues in the CCGA dataset. **B** ROC curves of PDIA3P1 for MES-GBM subtype prediction in CCGA. **C** Knockdown of PDIA3P1 in GSC20 and GSC267, overexpression of PDIA3P1 in GSC8-11. **D** Neurospheres formation assay revealed knockdown of PDIA3P1 reduced self-renewal capacity of GSC20 and GSC267. Scale bar, 200μm. The right panels were the quantification of sphere diameters. **E** The quantification of IF staining for SOX2 and CD44 in GSC20 (left panel) and GSC267 (right panel). Knockdown of PDIA3P1 resulted downregulation of CD44 and upregulation of SOX2 in GSC20 and GSC267.


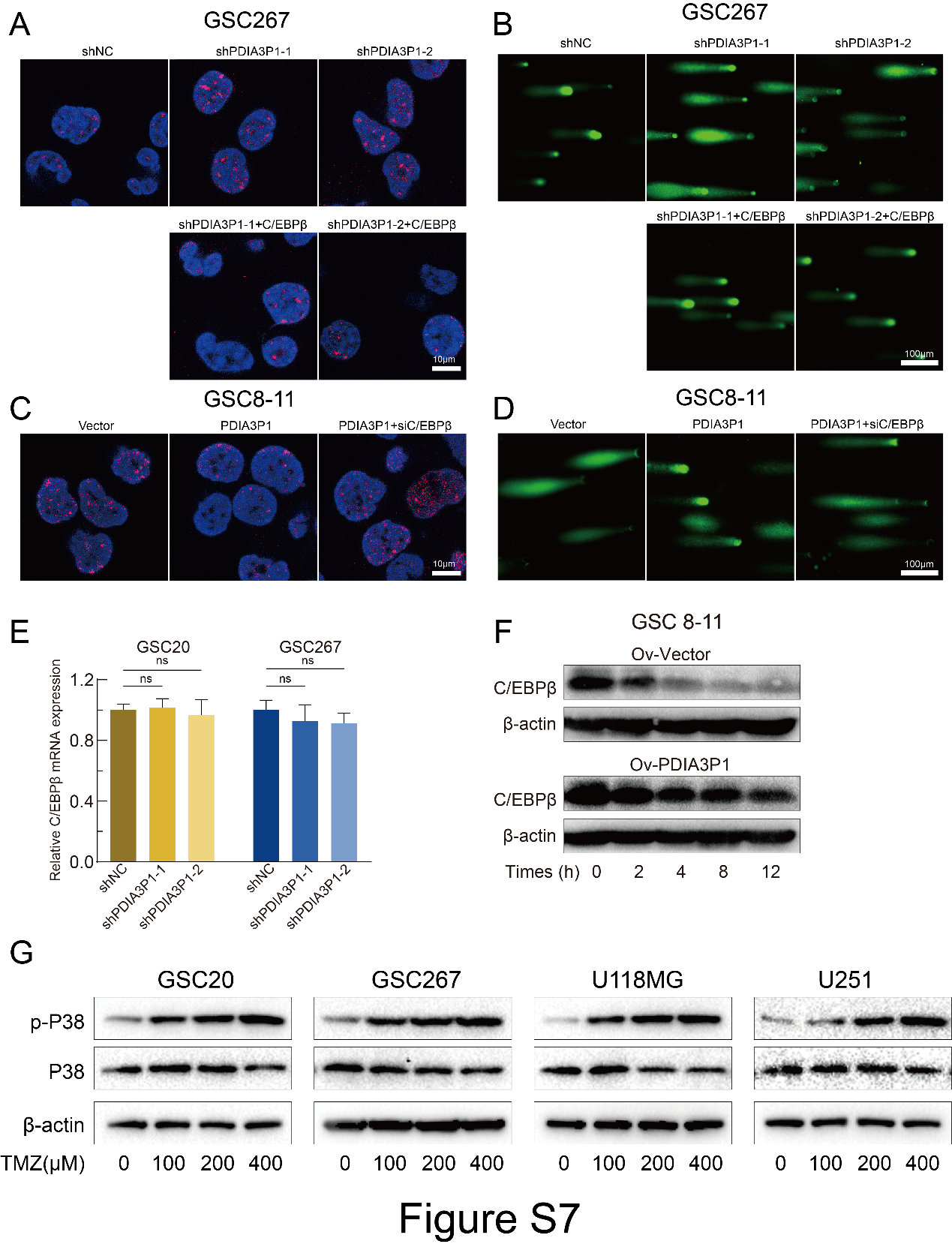


**Supplementary Figure 7**

**A B** Representative images of γ-H2AX IF staining (**A**. Scale bar, 10μm) and comet (**B**. Scale bar, 200μm) assays revealed DNA damage in GSC267. **C D** Representative images of γ-H2AX IF staining (**C**. Scale bar, 10μm) and comet (**D**. Scale bar, 200μm) assays revealed DNA damage in GSC8-11. **E** The expression of C/EBPβ mRNA detected by qPCR. **F** Western blotting analysis of C/EBPβ in GSC8-11 PDIA3P1 stable overexpressed and control cells after treatment with CHX (100 μg/ml) for indicated times. **G** Western blotting analysis of p-P38 and P38 expression with increasing concentrations of TMZ treatment.


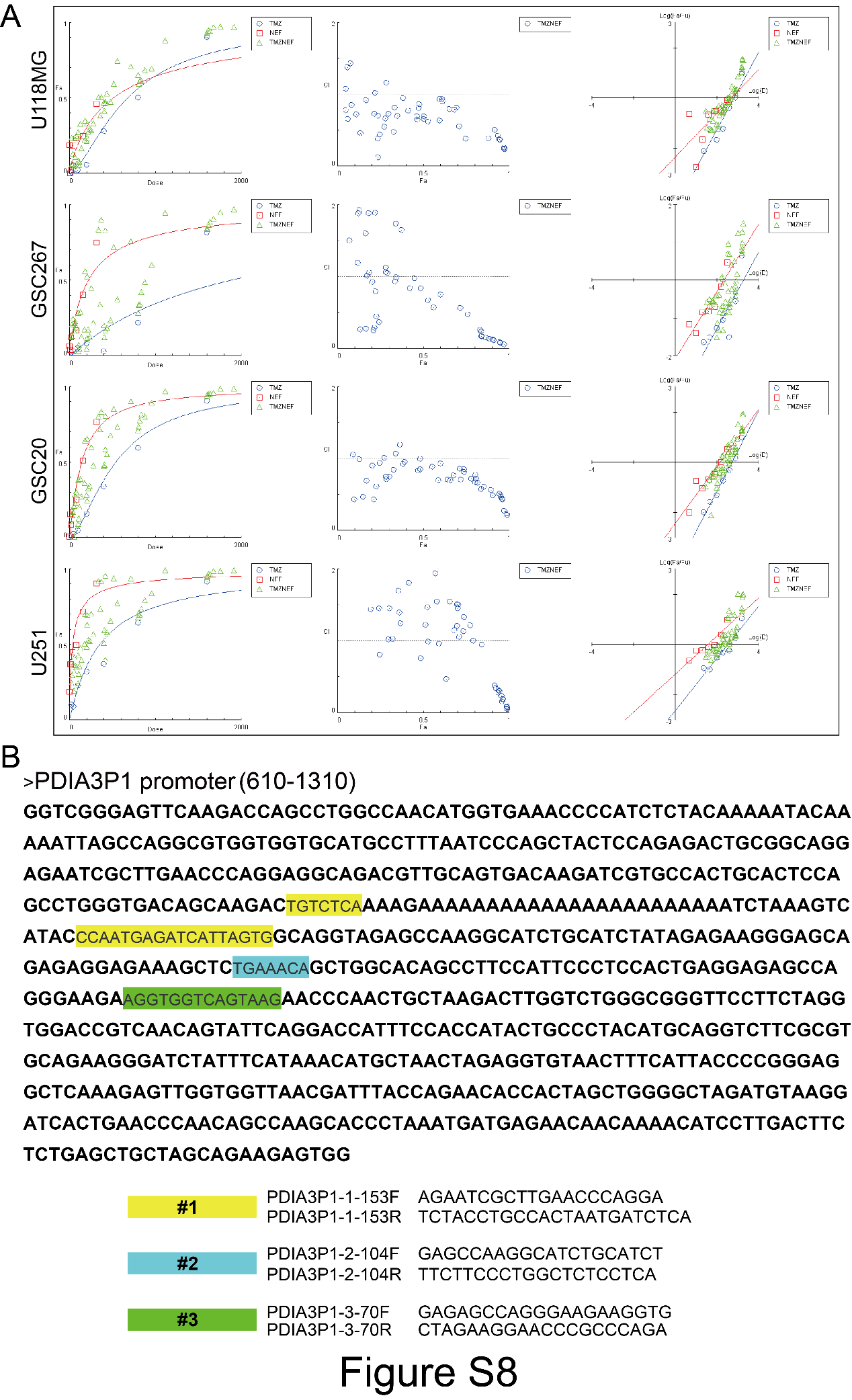


**Supplementary Figure 8**

**A** Visualization of the Fa-CI (fraction afected Combination Index) results obtained form CompuSyn for U118MG, GSC20, GSC267, and U251. **B** The detailed sequence of PDIA3P1 promoter (610-1310) and three predicted binding sites for JUN.


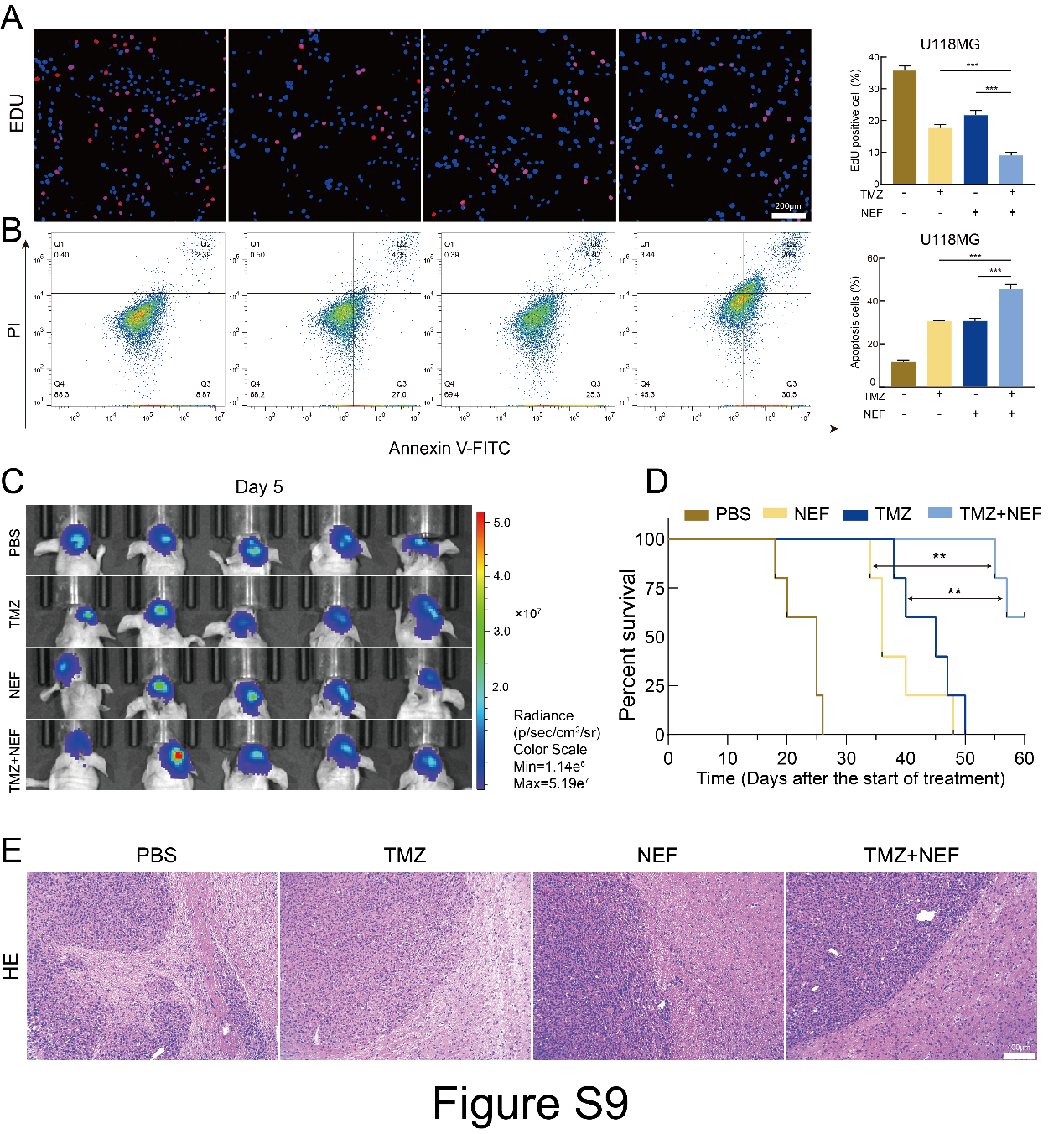


**Supplementary Figure 9**

**A B** EdU assay (**A**. Scale bar, 200μm) and apoptosis assay (**B**) showed that TMZ combined with NEF exhibited excellent anti-tumor cells effects, respectively. **C** Bioluminescence imaging of tumor growth on day five in GSC267 xenograft nude mice. **D** Kaplan–Meier visualized survival time for mice in different treatment groups. **E** Representative images of hematoxylin and eosin (H&E) staining in sections from GSC267 xenografts. Scale bar, 400μm
